# Alternating Current Electrolysis as Efficient Tool for the Direct Electrochemical Oxidation of Hydroxamic Acids for Acyl Nitroso Diels–Alder Reactions

**DOI:** 10.1002/anie.202107148

**Published:** 2021-08-08

**Authors:** Jan Fährmann, Gerhard Hilt

**Affiliations:** ^1^ Institut für Chemie Universität Oldenburg Carl-von-Ossietzky-Straße 9–11 26111 Oldenburg Germany

**Keywords:** alternating current, cycloaddition, Diels–Alder, electrochemistry, nitroso compounds

## Abstract

The acyl nitroso Diels–Alder reaction of 1,3‐dienes with electrochemically oxidised hydroxamic acids is described. By using alternating current electrolysis, their typical electro‐induced decomposition could be suppressed in favour of the 1,2‐oxazine cycloaddition products. The reaction was optimised using Design of Experiments (DoE) and a sensitivity test was conducted. A mixture of triethylamine/hexafluoroisopropanol served as supporting electrolyte in dichloromethane, thus giving products of high purity after evaporation of the volatiles without further purification. The optimised reaction conditions were applied to various 1,3‐dienes and hydroxamic acids, giving up to 96 % isolated yield.

## Graphical abstract

Hydroxamic acids were electrochemically oxidised to initiate acyl nitroso Diels–Alder reactions with 1,3‐dienes. By using alternating current, their often encountered electrochemically induced decomposition could be suppressed to generate the desired cycloaddition products in up to 96 % yield. The optimisation of this reaction was performed using the *Design of Experiments* approach.

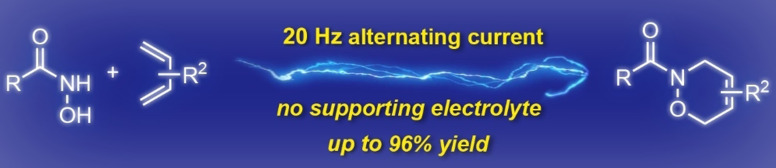

The Diels–Alder reaction is one of the most important cycloaddition reactions in organic chemistry and was honoured with the Nobel Prize in chemistry in 1950. Since then, many new discoveries in its research field were reported,^[1]^ for instance the hetero Diels–Alder reaction as a direct variation of its original but with hetero‐atoms like N, O and/or S in the diene or dienophile functionality.^[2]^ Especially nitroso compounds are interesting substrates for this reaction since the obtained 1,2‐oxazine unit is represented in biological active molecules^[3]^ or can be used as strategic intermediate in their total synthesis, for example, in dihydronarciclasine.^[4]^ Even hydroxamic acids are suitable substrates for the nitroso Diels–Alder reaction (NDA). Various oxidants can be used to generate a short‐lived acyl nitroso compound which can be trapped by 1,3‐dienes (Scheme 1).^[5]^ By electrochemical oxidation hydroxamic acids tend to decomposition under anodic conditions, thus forming acylium cation intermediates which react with various nucleophiles.^[6]^ Recently, Han published a method for the electrochemical cycloaddition of *N*‐phenylhydroxamic acids with alkenes for the synthesis of benzo‐1,2‐oxazines (Scheme 1).^[7]^ By *N*‐arylation, the anodic decomposition of the hydroxamic acid was suppressed and the cycloaddition products could be isolated in high yields.

**Scheme 1:**
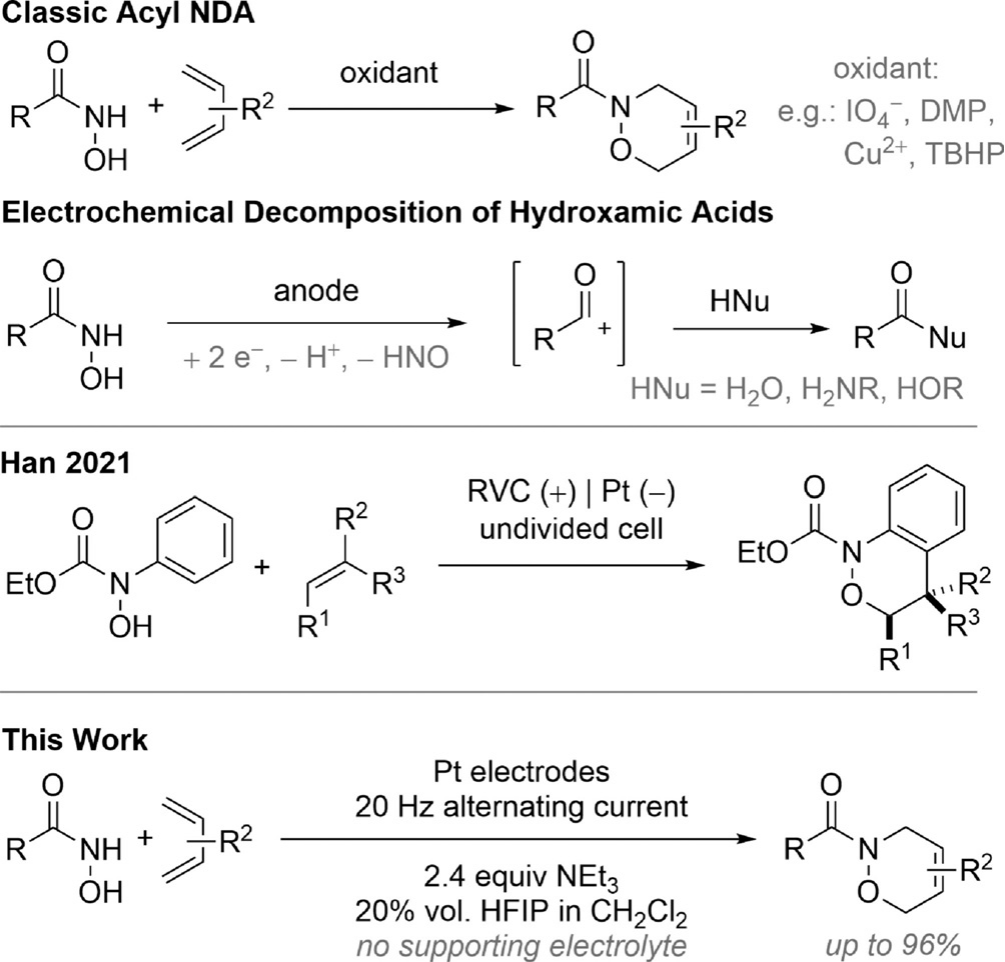
Previous work in the oxidation of hydroxamic acids.

In this article, we present the direct electrochemical oxidation of hydroxamic acids and the subsequent cycloaddition with 1,3‐dienes. The rarely used alternating current (AC) electrolysis^[8]^ enabled the classic acyl NDA by electro‐oxidation for the first time (Scheme 1). Firstly, the readily available benzohydroxamic acid (**1 a**) and 3.0 equiv of 1,3‐diene **2 a** were electrolysed in a 0.1 m solution of *n*Bu_4_NBF_4_ as supporting electrolyte in CH_2_Cl_2_.

The electrolysis was performed in an undivided cell with platinum electrodes at 10 mA direct current (DC, Table 1, entry 1). After 4.0 *F*, the conversion of the starting material was complete, but only benzoic acid was formed by anodic decomposition of the hydroxamic acid (Scheme 1). However, when the same reaction mixture was electrolysed under alternating current conditions (AC, 1.0 Hz) the cycloadduct **3 a** was obtained in 25 % yield. By addition of hexafluoroisopropanol (HFIP, 10 % vol.)^[9]^ the yield increased to 34 % (entry 2) and was further improved to 54 % by the addition of 2.4 equiv of pyridine as base (entry 3). Lowering the current of this reaction setup to 1.7 mA gave the maximum yield in the preliminary tests of 79 % (entry 4). Further optimisation including variation of the solvent, the supporting electrolyte and the electrode materials were performed (see Supporting Information). When these pre‐optimised reaction parameters were used in a DC electrolysis, the oxazine **3 a** was formed in 50 % yield (entry 4). It seemed that AC is not indispensable but has a positive impact on this reaction. For the optimisation of the numerical reaction parameters we applied the *DoE* approach.^[10]^ Here, we established two experimental designs, one for using AC and the other using DC to identify which type of current (AC/DC) is more suitable for this reaction. The DC experimental design was performed with a total of 21 experiments, whereas the AC design needed 31 experiments to be fully resolved due to two additional parameters (frequency and quiet time). Both designs were realised with high precision (R^2^=0.97 AC and R^2^=0.95 DC) and have low *p*‐values (both <0.01, Figure 1).

**Figure 1:**
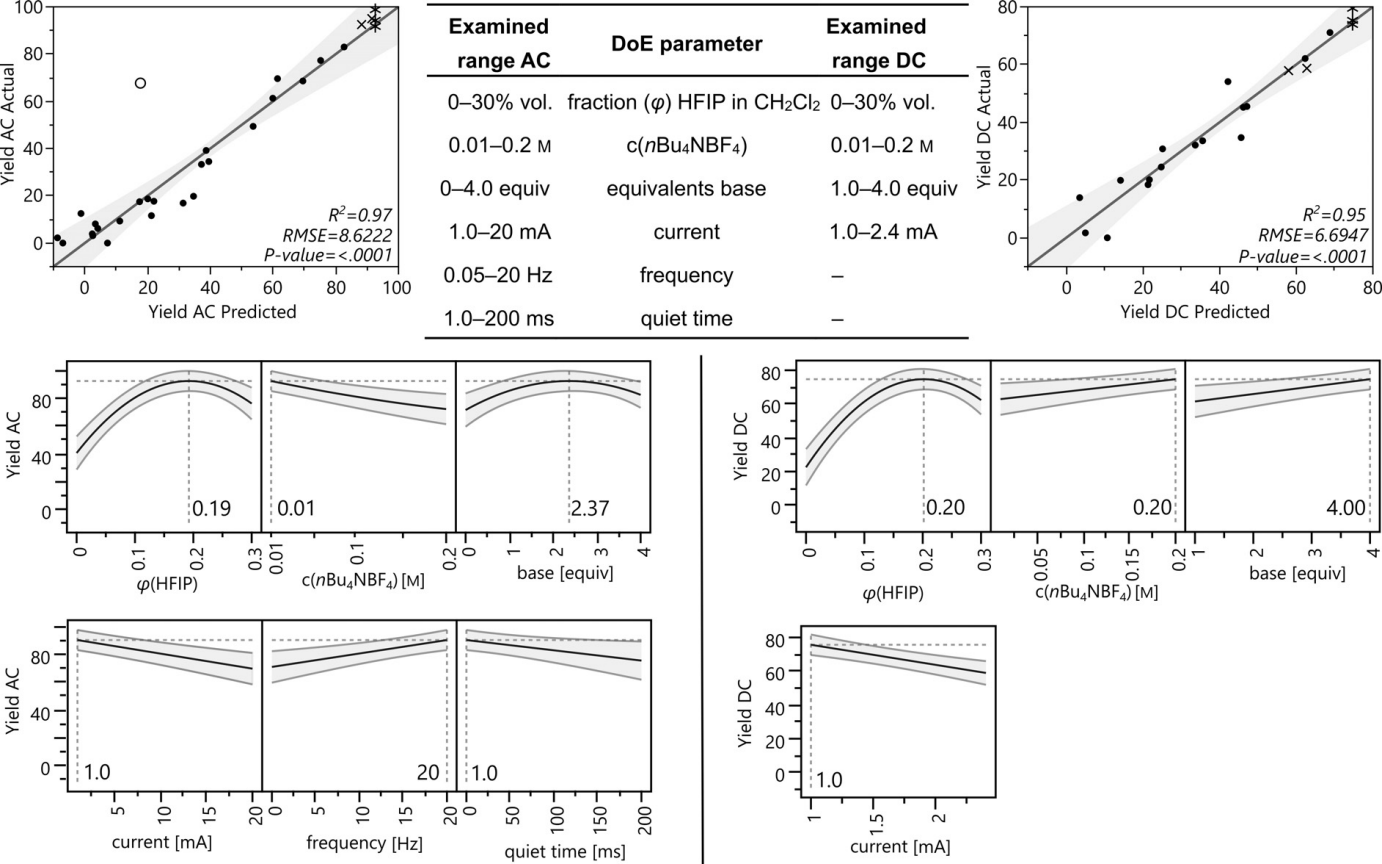
Results of the reaction optimisation by *DoE*. See Supporting Information for the full evaluation of the *DoE* results.

**Table 1:** Summarised optimisation experiments. 
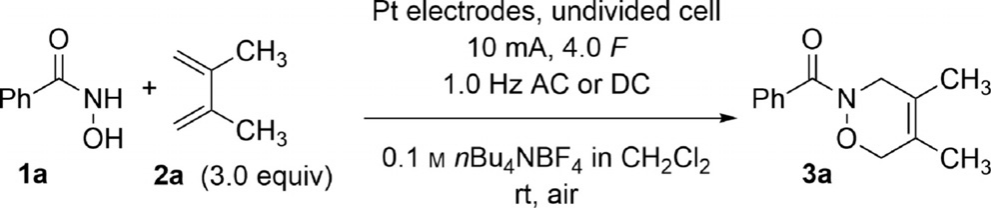

Entry	Yield^[a]^ AC	Changes from initial conditions	Yield^[a]^ DC
1	25 %	none	0 %
2	34 %	10 % vol. HFIP in CH_2_Cl_2_	0 %
3	54 %	entry 2 + 2.4 equiv pyridine	0 %
4	79 %	entry 3, but 1.7 mA	50 %

Entry	Yield^[a]^ AC	Changes from optimal conditions^[b]^	Yield^[a]^ DC
5	95 %	none	77 %
6	95 %	2.0 mA current	59 %
7	92 %	5.0 mA current	0 %
8	79 %	10 mA current	n.d.
9	*low conductivity*	entry 7, no supporting electrolyte	n.d.
10	91 %	entry 9, NEt_3_ instead of pyridine	n.d.
11	66 %	entry 10, 1.5 equiv 1,3‐diene **2 a**	n.d.

The electrolysis was performed with 0.25 mmol hydroxamic acid **1 a** in a total volume of 10 mL. Active surface of the Pt electrodes was 150 mm^2^ each. See Supporting Information for the complete list of optimisation experiments. [a] Determined by GC‐FID analysis of the crude reaction mixture with *n*‐dodecane as internal standard. [b] Optimal reaction conditions were carried out by *DoE* (Figure 1 and Supporting Information).

The optimum amount of HFIP is consistent for both models and should ideally be about 20 % vol. in CH_2_Cl_2_ (Figure 1). For the DC reaction, high electrolyte and base concentrations gave the best results, indicating that a high conductivity was necessary to obtain a high yield of the oxazine. However, when AC was applied, the lowest possible electrolyte concentration was best for a high generation of the desired product with an ideal base concentration of 2.4 equivalents. Both reactions run preferably at a low current, however the AC electrolysis setup proved to be less sensitive to a high current (compare entries 5–8, Table 1). When AC was applied, a frequency of 20 Hz with no quiet time is recommended.

Both reactions were performed at each optimised reaction conditions to yield 95 % at the optimised AC setup and 77 % at the DC reaction setup (entry 11). Both yields matched their model predictions of 93 % for AC and 75 % for DC and thus confirm the correctness of the models. Unfortunately, the current of 1.0 mA at the optimised reaction conditions is accompanied by long reaction times (107 h per mmol). Therefore, it was necessary to increase the applied current as far as reasonable with respect to the accompanying loss of oxazine yield. Doubling the current to 2.0 mA in the DC electrolysis already led to a relevantly reduced yield (59 %, entry 6), while 2.0 mA AC electrolysis did not reduce the yield. When the current was further increased to 5.0 mA, the yield at DC electrolysis dropped to 0 %, whereas it remained high at the AC reaction setup (92 %, entry 7). Even 10 mA AC could be applied and still 79 % of oxazine **3 a** were received. Overall, the AC electrolysis proved to be more suitable for this reaction due to a higher oxazine yield, higher economy in terms of electrolyte concentration and the higher applicable current, leading to drastically reduced reaction times. For the further investigations, 5.0 mA alternating current were used as the best compromise between reaction time and the product yield.

The results of the *DoE* proved, that the AC electrolysis is best performed at a low concentration of the supporting electrolyte, whereas omitting the electrolyte caused a too low conductivity for a successful electrolysis (entry 9). A stronger nitrogen‐base than pyridine is able to partially deprotonate HFIP, thus generating an electrolyte in situ and making the addition of an external electrolyte dispensable.^[9]^ Triethylamine proved to be a suitable base for this reaction (see Supporting Information), achieving a high conductivity without a supporting electrolyte (91 % yield, entry 10). Consequently, the separation from the product is omitted as well, because the solvent mixture can be removed under reduced pressure. As shown in the crude NMR spectrum (Figure S38 and S39), oxazines of high purity can be received without any purification steps if the 1,3‐diene is volatile too. When the excess of 1,3‐diene was reduced to 1.5 equiv, a lower yield was observed (66 %, entry 11) and therefore 3.0 equiv of the 1,3‐diene was kept as standard.

The optimised reaction conditions were used to investigate the substrate scope utilizing **1 a** and various 1,3‐dienes (Scheme 2). The oxazine **3 a** was isolated in 85 % yield on a larger scale of 0.50 mmol. Subsequently, when isoprene (**2 b**) was added, the unsymmetrical 1,3‐diene led to two regioisomers of the oxazines **3 b** which were isolated in 66 % as a 77:23 mixture of the distal and the proximal product. A possible reason for the lowered yield with respect to **2 a** might be the lower boiling point of isoprene and associated partial evaporation of the 1,3‐diene during the electrolysis. When the less volatile 1,4‐dimethyl substituted diene **2 c** was used, the product yield of **3 c** remained high at 79 %. Since penta‐1,3‐diene also tends to evaporation, 1‐cyclohexylbuta‐1,3‐diene (**2 d**) was used as model for a 1‐monosubstituted 1,3‐diene. The branched product **3 d** was isolated in a good yield of 86 % with a distal to proximal ratio of 35:65. However, the 1,1‐disubstituted diene **2 e** gave a moderate yield of 36 % as a single regioisomer (distal).^[11]^ The oxazine **3 f** was synthesised from cycloocta‐1,3‐diene (**2 f**) and isolated in 82 % yield, confirming that cyclic 1,3‐dienes can also be used for the electrochemical acyl NDA. On the other hand, electron‐rich 1,3‐dienes (**2 g**, **2 i** and **2 j**) with a phenyl‐ or oxygen‐substituent attached to the 1,3‐diene could not be used for this reaction, probably due to their low redox potential.^[12]^ However, aryl substituents are tolerated if the aromatic substituent is not directly bound to the 1,3‐diene functionality. Thus, the 1,3‐diene **2 h** afforded the oxaxine **3 h** in 67 % yield in a 50:50 distal to proximal ratio and even sorbic alcohol (**2 k**) could be used successfully to yield the distal‐configured oxazine **3 k** in 68 % as a single regioisomer.

**Scheme 2:**
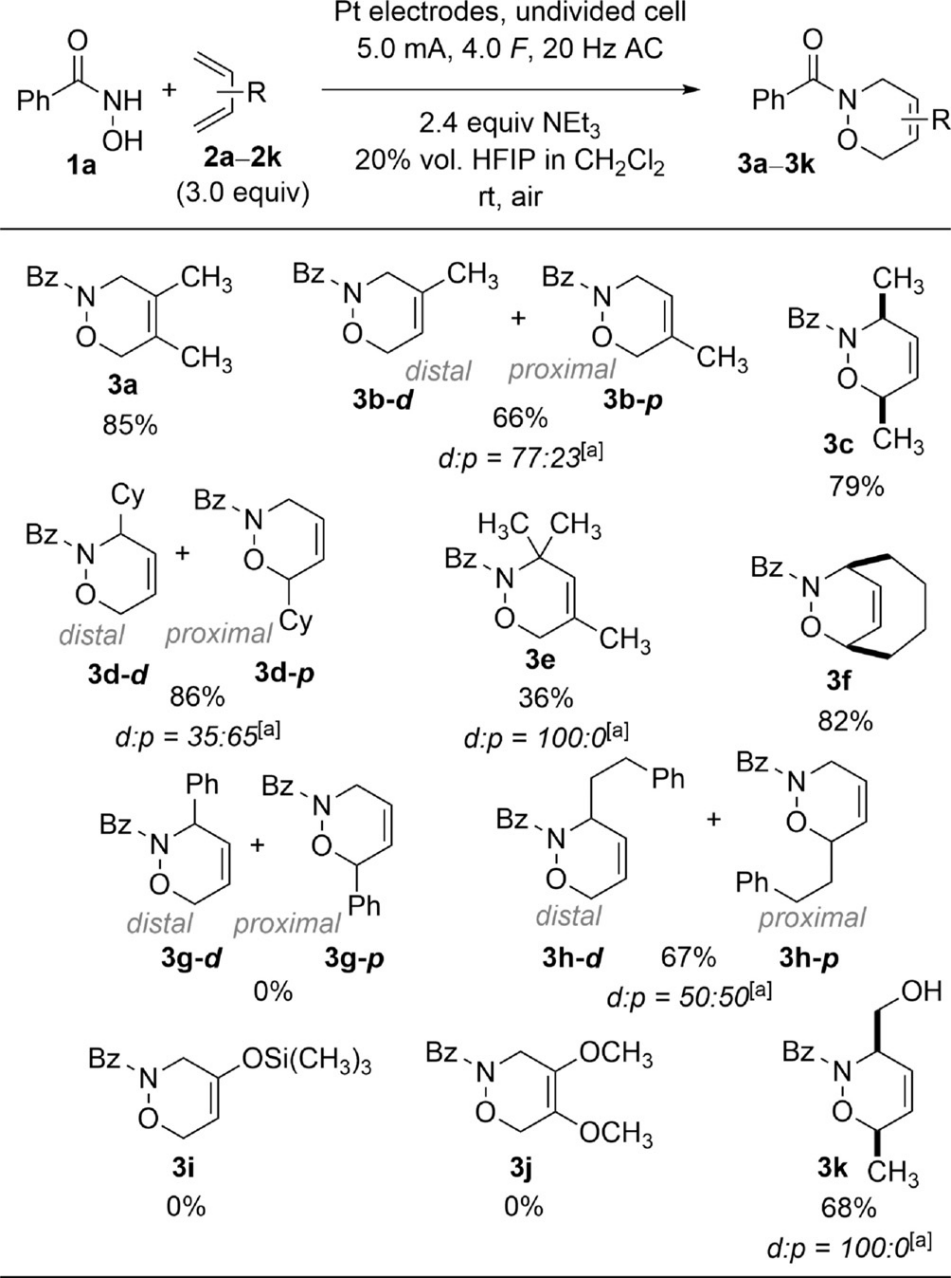
Substrate scope with varied 1,3‐dienes. The electrolysis was performed with 0.50 mmol hydroxamic acid **1 a** in a total volume of 10 mL. The active surfaces of the Pt electrodes were 150 mm^2^ each. [a] Determined by integration of separated ^1^H NMR signals of the crude reaction mixture. Bz=benzoyl, Cy=cyclohexyl.

For the variation of the hydroxamic acids **1 a**–**1 k**, diene **2 a** was chosen as reactant due to its commercial availability and the avoidance of distal‐proximal product mixtures (Scheme 3). In the first step, the electronic influence on aromatic hydroxamic acids was investigated. When an electron‐withdrawing nitro‐substituent was included in the aromatic moiety, only traces of the oxazine **4 a** were observed by GC‐MS analysis. Since the oxazine **5 a** with an electron‐withdrawing −CF_3_ group was isolated in a high yield of 89 %, the electrochemical reduction of the nitro group is a plausible explanation for the low yield of **4 a**. Electron‐rich arenes **1 d** and **1 e** afforded similar yields compared to the unsubstituted benzohydroxamic acid **1 a** (81 % for **6 a** and 77 % for **7 a**). In contrast, the aliphatic hydroxamic acids gave the desired products **8 a**–**11 a** in moderate yields ranging from 41 % to 64 %. In these cases, steric hindrance seems not to be relevant because the *tert*‐butyl substituted product **10 a** was formed in 54 %. The reason might be associated with less effective deprotonation of the hydroxamic acids prior to oxidation (see below). The hydroxyl carbamates **1 j** and **1 k** represent the hydroxamic acid of the common protection groups *Boc* (**1 j**) and *Cbz* (or *Z*, **1 k**). These substrates are of special interest since cleavage of the amide bond in the product can be realised easily to give the free oxazine for further manipulations.^[13]^ These hydroxamic acids were used to generate the highly interesting oxazines **12 a** and **13 a** in good to excellent yields (75 % and 96 %, respectively).

**Scheme 3:**
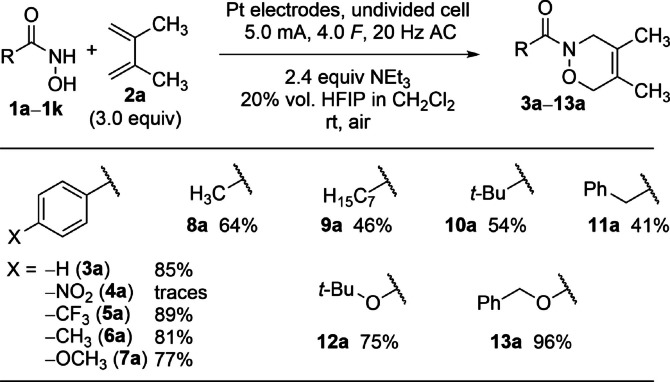
Substrate scope with varied hydroxamic acids. The reaction conditions are consistent to Scheme 2.

To identify the most influential parameters of the reaction conditions and thus support the reproducibility of this reaction, we performed a sensitivity test.^[14]^ The results of these manipulations are summarised in Figure 2. A large electrode surface led to a significant loss in productivity. Also, the yield was lowered noticeably when the amount of base varied from 2.4 equivalents. In particular, a low concentration of base resulted in a greater loss of the product than a high concentration. Little fluctuations in room temperature did not have a significant impact on the product yield. The reaction seemed to be not sensitive in terms of inert conditions and applied current. The NDA reaction could be performed under air without dried solvents since water and oxygen and applied current had little impact (±3–5 % yield) when differed by ±1.0 *F*.

**Figure 2:**
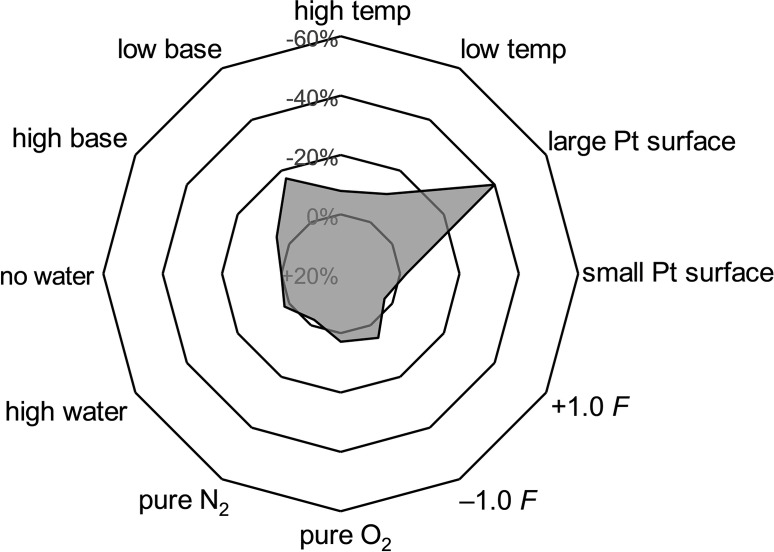
Sensitivity test of the electrochemical NDA reaction.

In a brief survey of electroanalytical control experiments by cyclic voltammetry (CV, see Supporting Information) the onset potential for the oxidation of **1 a** shifted from +1.2 V to +0.6 V (Pt electrodes vs. Ag/AgCl) by addition of NEt_3_.^[15]^ The deprotonation of **1 a** seems to lower the oxidation potential considerably for the transformation from **A** to a short‐lived intermediate **B** (Scheme 4). The irreversibility of the electrochemical oxidation also reveals that a fast follow‐up deprotonation and a second oxidation towards the acyl nitroso compound **C** are likely to happen.^[16]^ Upon addition of the 1,3‐diene to this solution, only relatively small changes were observed in CV. However, a drastic reduction of the peak current and a shift of the peak potential was observed when additional CVs were measured shortly thereafter. Thus, electrode fouling is likely to occur under the increasing oxidation potential of the CV, which might be similar to the DC electrolysis over a short period of time. Accordingly, only small current densities are acceptable when preparative DC electrolysis were used for the NDA reaction to keep the working electrode passivation on a low level. In contrast, AC electrolysis has been applied in the past successfully to remove passivating films from working electrodes^[17]^ to set higher current densities and minimise electrode fouling. From our perspective, this seems to be a key point why AC gave considerably better results over DC electrolysis in this case. The need of 2.4 equivalents base can be rationalised by the double deprotonation of the hydroxamic acid towards the acyl nitroso intermediate and the formation of a HFIP‐base electrolyte. Also, HFIP as co‐solvent might stabilise the radical intermediate **B**, in analogy to the suggestions made by Waldvogel.^[18]^

**Scheme 4:**
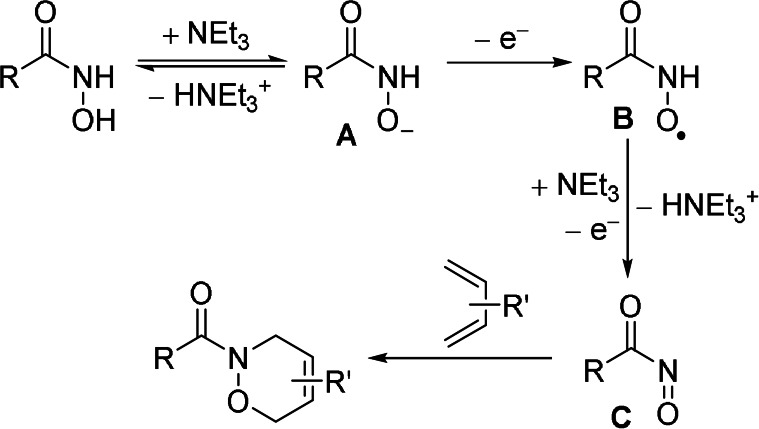
Proposed reaction mechanism.

In conclusion, we optimised the electrochemical acyl NDA of benzohydroxamic acid (**1 a**) with 2,3‐dimethylbutadiene (**2 a**) using *DoE* to obtain the desired 1,2‐oxazine **3 a** in 95 % yield. By using two different experimental designs we compared the product formation using AC and DC, in which alternating current electrolysis gave the best results and prove to be much more robust at high current than the DC electrolysis. The supporting electrolyte consisted of a volatile solvent‐base mixture so that the reaction products can be isolated in high purity without purification after evaporation of the solvent. Various hydroxamic acids and 1,3‐dienes were applied successfully whereas the application of electron‐rich 1,3‐dienes is limited by their lower oxidation potential.^[12]^

## Conflict of interest

The authors declare no conflict of interest.

## Supporting information

As a service to our authors and readers, this journal provides supporting information supplied by the authors. Such materials are peer reviewed and may be re‐organized for online delivery, but are not copy‐edited or typeset. Technical support issues arising from supporting information (other than missing files) should be addressed to the authors.

Supporting Information
