# Supplementary material for: Alternating Current Electrolysis as Efficient Tool for the Direct Electrochemical Oxidation of Hydroxamic Acids for Acyl Nitroso Diels–Alder Reactions
Source: Angew Chem Int Ed Engl. 2021 Aug 8;60(37):20313–7. doi: 10.1002/anie.202107148 (PMC8456936; doi:10.1002/anie.202107148)
Supplement: Supplementary file 1 — Supporting Information [file ANIE-60-20313-s001.pdf]

Angewandte Chemie
Eine Zeitschrift der Gesellschaft Deutscher Chemiker

Supporting Information

Alternating Current Electrolysis as Efficient Tool for the Direct Electrochemical Oxidation of Hydroxamic Acids for Acyl Nitroso Diels–Alder Reactions

*Jan Fährmann and Gerhard Hilt**

anie_202107148_sm_miscellaneous_information.pdf

Content

1 General information ... 2

2 Optimization table ... 4

3 Design of experiments (DoE) ... 5

3.1 DC DoE raw data ... 5

3.2 Calculated DC DoE model ... 6

3.3 AC DoE raw data ... 7

3.4 Calculated AC DoE model ... 8

4 Sensitivity test[1] ... 9

5 Experimental section ... 10

5.1 General procedure for the synthesis of hydroxamic acids ... 10

5.2 General procedure for the electrochemical acyl-NDA ... 10

6 Analytical section ... 11

6.1 Analytical data of synthesized hydroxamic acids ... 11

6.2 Analytical data of synthesized 1,2-oxazines ... 12

6.3 NMR-Spectra of the synthesized 1,2-oxazines ... 20

6.4 NMR spectrum of the crude reaction product ... 32

7 Mechanistic investigations ... 33

7.1 Cyclic voltammetry ... 34

8 References ... 35

1 General information

All reactions were carried out under atmospheric conditions. The used solvents were purchased in high purity (HPLC grade). Commercially available chemicals were used without further purification. Electrolysis were executed in undivided under constant current. Therefore, a laboratory power supply from *Aim TTi MX100T Triple Output Multi-Range DC Power Supply* (35 V, 3 A) was used. As electrode materials platinum plates (dimensions 35 mm x 10 mm x 0.5 mm, purity min. 99.95%), glassy carbon plates (dimensions 35 mm x 10 mm x 0.5 mm), carbon roving (6 k, 400 tex), copper (Cu-DHP according to ENCW024A, dimensions 35 mm x 10 mm x 0.5 mm) and stainless steel (material number 1.4571 according to EN10027-2, dimensions 35 mm x 10 mm x 0.5 mm) were utilised. The switching of the electrode polarisation was accomplished by a custom-made device built by the electronics workshop of the University of Oldenburg. For interrupted direct current, a custom-made timing relay device was used with a *CT-MFE* unit from *KlingerBorn*. *Design of Experiments* was performed using JMP 16 software package by SAS (version 16.0.0. SAS Institute Inc., Cary, NC, 2021).

NMR spectra were recorded on a *Bruker Avance III* spectrometer utilising pre-set pulse programs. If not stated otherwise, the measurements were performed at room temperature. The chemical shifts are given in parts per million (ppm). Calibration was done by referring to the residual solvent signal ($CDCl_3$: ^{1}H NMR 7.26 ppm, ^{13}C NMR 77.16 ppm; DMSO-d_6: ^{1}H NMR 2.50 ppm, ^{13}C NMR 39.52 ppm) in relation to tetramethylsilane.
HR-MS (High resolution mass spectra) were recorded on a *Thermo Scientific DFS spectrometer* using electron ionization (EI).
GC-MS measurements were performed on a *Shimadzu GCMS-QP2020*. As capillary column an *Optima 5 HT* from *Macherey-Nagel* was used (length 30 m, inner diameter 0.25 mm, film thickness 0.25 µm). The ionisation was accomplished by electron impact (EI) with an energy of 70 eV.
GC-FID measurements were carried out on a *Shimadzu GC-2010 Plus* gas chromatograph with an *Optima 5 MS* capillary column from *Macherey-Nagel* (length 15 m, inner diameter 0.25 mm, film thickness 0.25 µm).
CV (Cyclic voltammetry) measurements were carried out on a *CH Instruments* cell stand and a *CH Instruments 620A* electrochemical analyzer using a platinum disk working electrode (2.0 mm diameter) and platinum wire counter electrode (0.5 mm diameter). Potentials were referred to a saturated Ag/AgCl (3 M KCl) reference electrode. Cyclic voltammograms were measured on *CHI Version 2.02* software.

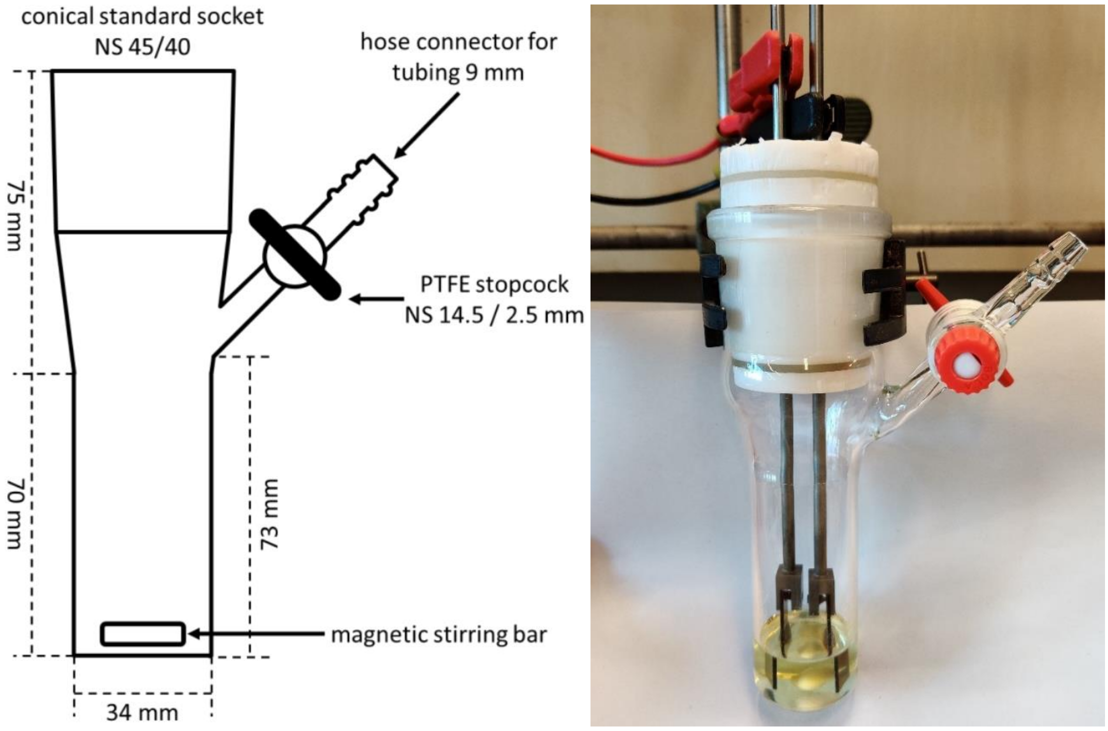

Figure S1: (left) Sketch of the undivided cells that were used. (right) Example of an electrochemical cell-setup.

2 Optimization table

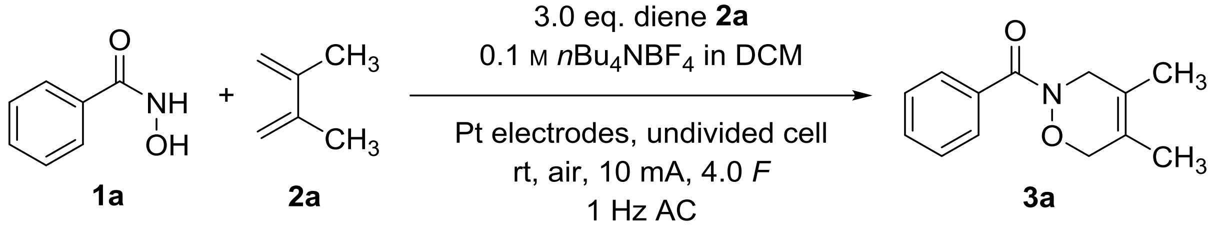

1a **2a** **3a**

entry	changes to initial conditions	yield*
	supporting electrolyte	
**1**	**none**	**25%**
2	Me_4NBF_4	22%
3	*n*Bu_4NPF_6	6%
4	Et_4NOTs	11%
5	*n*Bu_4NClO_4	6%
	Solvent	
6	MeCN	20%
7	DMF	17%
8	acetone	25%
9	THF	17%
10	DMSO	3%
11	1,2-dichloroethane	22%
12	1,2-dimethoxyethane	22%
13	DCM:TFE 1.0:2.5[a]	31%
14	DCM:EtOH 1.0:2.5	12%
**15**	**DCM:HFIP 10:1.0**[b]	**34%**
16	DCM:HFIP 1.0:1.3[b]	25%
17	acetone : HFIP 10:1[][b]	9%
18	DCM:TFE 10:1.0[a]	26%
19	DCM:TFE 1:1[a]	28%
	electrolyte concentration (*n*Bu_4NBF_4)	
20	0.013 M	31%
21	0.025 M	29%
22	0.05 M	29%
23	0.20 M	29%
	addition of base	
24	2.4 eq. lutidine	51%
25	13.4 eq. lutidine	39%
26	2.4 eq. *t*Bu_2Py[c]	51%
**27**	**2.4 eq. pyridine**	**54%**
28	2.4 eq. NEt_3	53%
29	2.4 eq. piperidine	51%

entry	changes to initial conditions	yield*
	electrode material	
30	graphite	43%
31	glassy carbon	31%
32	Cu	0%
33	stainless steel	3%
34	Ni	2%
35	Al	0%
	AC frequency	
36	DC	0%
37	interrupted DC[d]	0%
38	0.015 Hz	45%
39	0.050 Hz	51%
40	0.100 Hz	53%
41	10.0 Hz	57%
42	100 Hz	40%
	current	
43	10 mA	40%
44	5.0 mA	73%
45	**1.7 mA**	**79%**
	quiet time	
46	100 ms	60%
47	1000 ms	46%
51	no current	0%

The electrolysis was performed with 0.25 mmol hydroxamic acid **1a** in a total volume of 10 mL. Active surface of the Pt electrodes was 150 mm² each. Changes from highlighted entries were taken for the following experiments. * Determined by GC-FID analysis of the crude reaction mixture with *n*-dodecane as internal standard. [a] TFE = 1,1,1-trifluoroethanol. [b] HFIP = 1,1,1,3,3,3-hexafluoroisopropanol. [c] *t*Bu_2Py = 2,6-di-*tert*-butyl-pyridine. [d] applied as 0.1 s pulse followed by 0.1 s quiet time without switching of the electrodes polarity.

3 Design of experiments (DoE)

Both designs were calculated in a D-optimal plan and include repeated experiments to determine the *lack of fit*.

3.1 DC DoE raw data

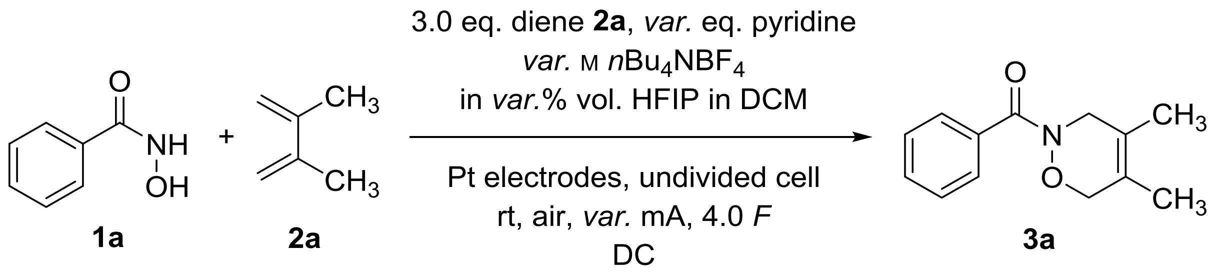

Entry / marking in DoE model	prop(HFIP) [v/v]	c(electrolyte) [mol/L]	base [Eq]	current [mA]	yield* [at 4 *F*]
1 / •	0	0,2	4	1,7	19,9%
2 / •	0,15	0,2	2,5	1	71,1%
3 / •	0,3	0,105	2,5	1,7	32,1%
4 / •	0	0,01	4	1	0,0%
5 / •	0	0,2	1	2,4	24,5%
6 / •	0,3	0,01	4	1,7	54,1%
7 / •	0	0,01	1	1,7	18,4%
8 / •	0	0,105	1	1	33,6%
9 / •	0,3	0,2	4	2,4	34,7%
10 / •	0,3	0,01	1	2,4	1,7%
11 / •	0,141	0,1012	4	2,4	45,5%
12 / •	0,3	0,2	4	1	62,1%
13 / •	0	0,01	2,5	2,4	13,9%
14 / •	0,3	0,2	1	1,7	30,8%
15 / •	0,3	0,01	1	1	20,1%
16 / •	0,15	0,01	1	1,7	45,2%
17 / ∗	0,2	0,2	4	1	73,9%
18 / ∗	0,2	0,2	4	1	79,5%
19 / ∗	0,2	0,2	4	1	75,3%
20 / ×	0,2	0,2	4	2	58,7%
21 / ×	0,2	0,2	4	2,4	57,9%

The electrolysis was performed with 0.25 mmol hydroxamic acid **1a** in a total volume of 10 mL. Active surface of the Pt electrodes was 150 mm² each. * Determined by GC-FID analysis of the crude reaction mixture with *n*-dodecane as internal standard. Entries 1–16 represent the suggested experiments for a fully resolved DoE analysis. Entries 17–21 represent additional experiments of the optimal reaction conditions and for the determination of the *lack of fit*.

3.2 Calculated DC DoE model

Response Yield DC [at 4 F]

Actual by Predicted Plot

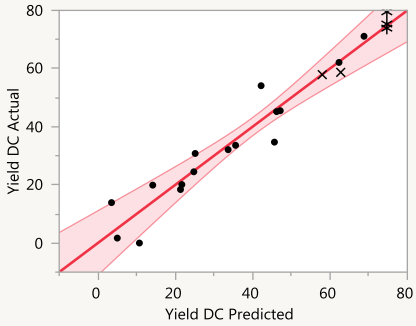

RMSE=6,6947 RSq=0,95 PValue=<,0001

Effect Summary

Source	LogWorth	PValue	
Prop(HFIP)*Prop(HFIP)	5,932	0,00000	
Prop(HFIP)*Base [Eq]	4,310	0,00005	
Current [mA](1,2,4)	3,408	0,00039	
Prop(HFIP)(0,0,3)	3,081	0,00083	^
c(Electrolyte) [mol/L](0,01,0,2)	2,149	0,00710	
Base [Eq](1,4)	0,695	0,20194	^

Lack Of Fit

Source	DF	Sum of Squares	Mean Square	F Ratio
Lack Of Fit	12	610,48089	50,8734	5,9898
Pure Error	2	16,98667	8,4933	**Prob > F**
Total Error	14	627,46756		0,1518
				**Max RSq**
				0,9986

Residual by Predicted Plot

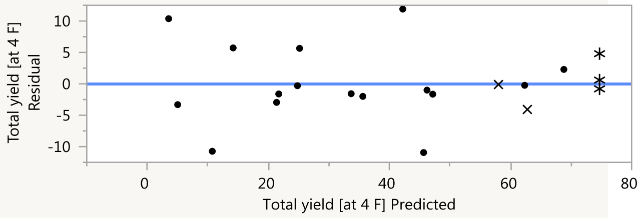

Studentized Residuals

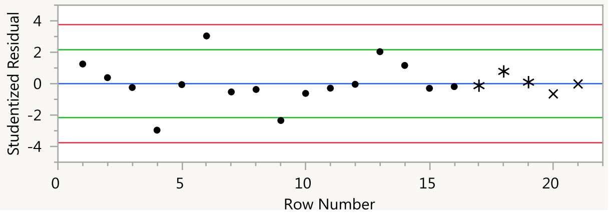

Externally studentized residuals with 95% simultaneous limits (Bonferroni) in red, individual limits in green.

Prediction Profiler

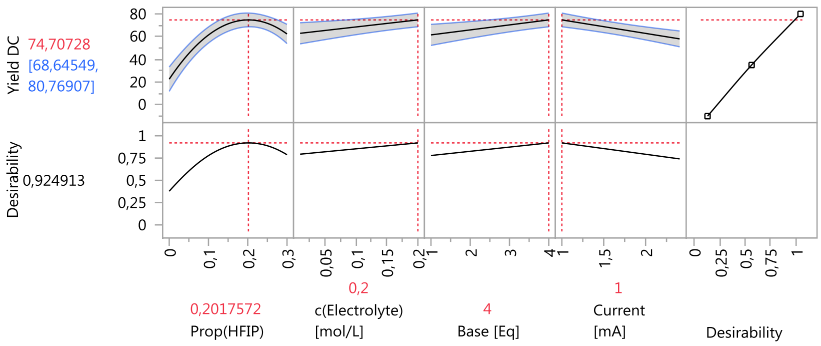

3.3 AC DoE raw data

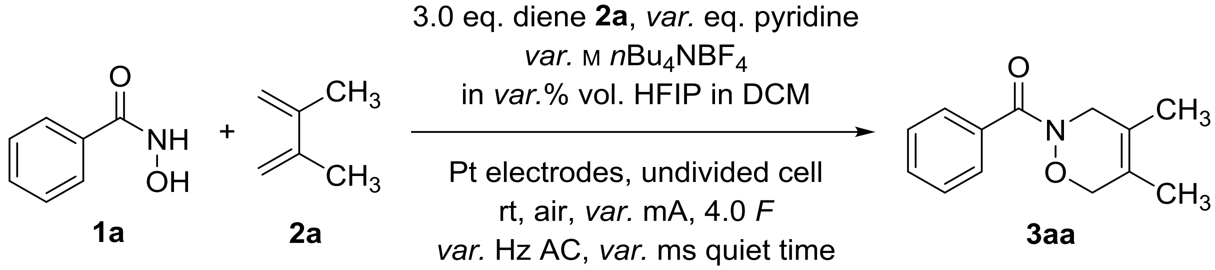

entry	prop(HFIP) [v/v]	c(electrolyte) [mol/L]	base [Eq]	current [mA]	frequency [Hz]	pause [ms]	yield* [at 4 *F*]
1 / •	0	0,2	4	20	0,05	200	3,1%
2 / •	0,3	0,105	4	10,5	0,05	200	39,2%
3 / •	0	0,2	0	1	10,025	200	18,7%
4 / •	0,15	0,01	4	10,5	20	200	49,4%
5 / o	0,3	0,2	4	1	0,05	1	67,7%
6 / •	0,3	0,105	0	20	0,05	1	0,0%
7 / •	0	0,2	2	20	0,05	1	3,9%
8 / •	0	0,01	2	10,5	10,025	1	11,6%
9 / •	0	0,105	0	20	20	200	2,2%
10 / •	0,3	0,01	0	1	0,05	1	16,9%
11 / •	0	0,01	0	20	0,05	200	6,2%
12 / •	0	0,2	0	10,5	20	100,5	9,3%
13 / •	0,15	0,105	4	1	10,025	1	61,3%
14 / •	0,3	0,2	4	1	20	1	33,3%
15 / •	0,3	0,2	0	10,5	10,025	100,5	12,5%
16 / •	0,15	0,2	0	20	20	1	19,8%
17 / •	0	0,01	4	1	20	100,5	17,5%
18 / •	0,3	0,105	2	1	20	200	–
19 / •	0	0,105	4	20	20	1	8,1%
20 / •	0,3	0,2	2	20	20	200	0,0%
21 / •	0,3	0,01	4	20	10,025	100,5	34,5%
22 / •	0	0,01	4	1	0,05	200	17,7%
23/ •	0,15	0,105	2	1	0,05	100,5	68,6%
24 / •	0,3	0,01	2	1	20	1	77,3%
25 / •	0,24	0,01	4	1	0,05	1	69,6%
26 / •	0,18	0,9	4	1	0,05	100	83,0%
27 / ⚹	0,2	0,01	2,4	1	20	1	91,7%
28 / ⚹	0,2	0,01	2,4	1	20	1	98,8%
29 / ⚹	0,2	0,01	2,4	1	20	1	94,3%
30 / ×	0,2	0,01	2,4	2	20	1	95,0%
31 / ×	0,2	0,01	2,4	5	20	1	92,5%

The electrolysis was performed with 0.25 mmol hydroxamic acid **1a** in a total volume of 10 mL. Active surface of the Pt electrodes was 150 mm² each. * Determined by GC-FID analysis of the crude reaction mixture with *n*-dodecane as internal standard. Entries 1–26 represent the suggested experiments for a fully resolved DoE analysis. Entries 27–31 represent additional experiments of the optimal reaction conditions and for the determination of the *lack of fit*.

3.4 Calculated AC DoE model

Response Yield AC [at 4 F]

Actual by Predicted Plot

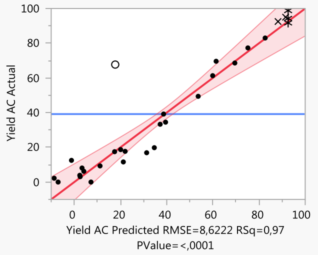

Effect Summary

Source	LogWorth	PValue	
Prop(HFIP)*Prop(HFIP)	5,612	0,00000	
Current [mA](1,20)	3,583	0,00026	
c(Electrolyte) [mol/L]*c(Electrolyte) [mol/L]	3,400	0,00040	
Prop(HFIP)(0,0,3)	2,984	0,00104	^
Base [Eq]*Base [Eq]	2,766	0,00172	
Frequency [Hz]*Pause [ms]	2,629	0,00235	
c(Electrolyte) [mol/L](0,01,0,2)	2,620	0,00240	^
Prop(HFIP)*c(Electrolyte) [mol/L]	2,510	0,00309	
Prop(HFIP)*Base [Eq]	1,354	0,04427	
Base [Eq](0,4)	1,036	0,09207	^
Frequency [Hz](0,05,20)	0,424	0,37657	^
Pause [ms](1,200)	0,066	0,85872	^

Lack Of Fit

Source	DF	Sum of Squares	Mean Square	F Ratio
Lack Of Fit	14	1163,6719	83,1194	6,4417
Pure Error	2	25,8067	12,9033	Prob > F
Total Error	16	1189,4786		0,1423
				Max RSq
				0,9993

Residual by Predicted Plot

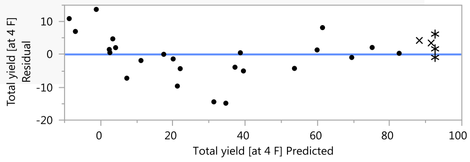

Studentized Residuals

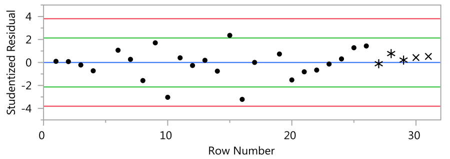

Externally studentized residuals with 95% simultaneous limits (Bonferroni) in red, individual limits in green.

Parameter Estimates

Term	Estimate	Std Error	t Ratio	Prob>|t|
Intercept	61,02744	4,522568	13,49	<,0001 *
Prop(HFIP)(0,0,3)	8,5331188	2,134702	4,00	0,0010 *
c(Electrolyte) [mol/L](0,01,0,2)	-7,781304	2,161431	-3,60	0,0024 *
Base [Eq](0,4)	3,9826779	2,222525	1,79	0,0921
Current [mA](1,20)	-10,35522	2,22203	-4,66	0,0003 *
Frequency [Hz](0,05,20)	1,8625216	2,047802	0,91	0,3766
Pause [ms](1,200)	0,4248671	2,348733	0,18	0,8587
Prop(HFIP)*Prop(HFIP)	-31,6714	4,450359	-7,12	<,0001 *
c(Electrolyte) [mol/L]*c(Electrolyte) [mol/L]	1,4544945	0,326381	4,46	0,0004 *
Base [Eq]*Base [Eq]	-15,01016	3,993327	-3,76	0,0017 *
Prop(HFIP)*c(Electrolyte) [mol/L]	-8,396433	2,412712	-3,48	0,0031 *
Prop(HFIP)*Base [Eq]	5,3915577	2,469631	2,18	0,0443 *
Frequency [Hz]*Pause [ms]	-7,865348	2,178711	-3,61	0,0023 *

Effect Tests

Source	Nparm	DF	Sum of Squares	F Ratio	Prob > F
Prop(HFIP)(0,0,3)	1	1	1187,8946	15,9787	0,0010 *
c(Electrolyte) [mol/L](0,01,0,2)	1	1	963,5154	12,9605	0,0024 *
Base [Eq](0,4)	1	1	238,7228	3,2111	0,0921
Current [mA](1,20)	1	1	1614,5656	21,7180	0,0003 *
Frequency [Hz](0,05,20)	1	1	61,4984	0,8272	0,3766
Pause [ms](1,200)	1	1	2,4326	0,0327	0,8587
Prop(HFIP)*Prop(HFIP)	1	1	3765,1394	50,6459	<,0001 *
c(Electrolyte) [mol/L]*c(Electrolyte) [mol/L]	1	1	1476,4205	19,8597	0,0004 *
Base [Eq]*Base [Eq]	1	1	1050,3583	14,1287	0,0017 *
Prop(HFIP)*c(Electrolyte) [mol/L]	1	1	900,3584	12,1110	0,0031 *
Prop(HFIP)*Base [Eq]	1	1	354,3245	4,7661	0,0443 *
Frequency [Hz]*Pause [ms]	1	1	968,8877	13,0328	0,0023 *

Prediction Profiler

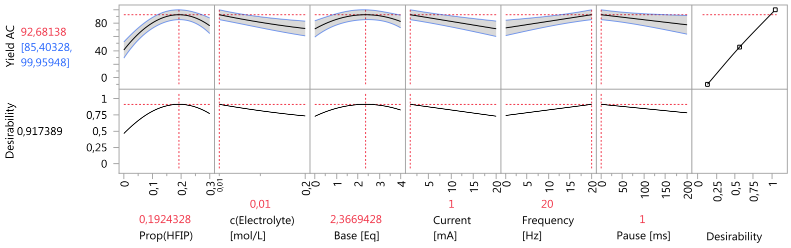

The AC design has one outlier (marked “o”) for the parameter settings 30% vol. HFIP, 0.2 M electrolyte concentration, 4.0 equiv. pyridine, 1.0 mA current, 0.05 Hz and 1 ms quiet time. The actual yield of 68% was significantly higher than its model-prediction of 18%, it was therefore excluded from the experimental design. Interestingly, it matched the predicted yield for these parameter settings in the DC design very well (predicted 63%) and resembles the optimum reaction conditions for the DC design in most settings. The low frequency (20 s electrolysis time before polarity switching) may favour a DC-like reaction course for this experiment.

4 Sensitivity test[1]

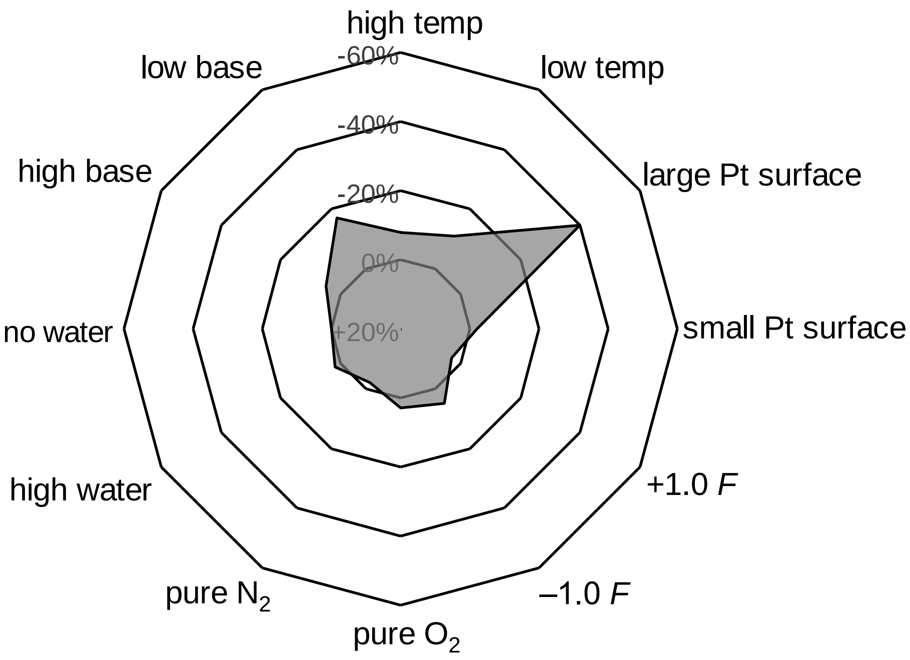

entry	change	to value	change in yield
1	high temp	40 °C instead of rt	–8%
2	low temp	0 °C instead of rt	–11%
3	large Pt surface	300 mm² instead of 150 mm²	–40%
4	small Pt surface	75 mm² instead of 150 mm²	-2%
5	more current	5 *F* instead of 4 *F*	+3%
6	Less current	3 *F* instead of 4 *F*	–5%
7	pure O_2	100% O_2 atmosphere instead of air	–3%
8	pure N_2	100% N_2 atmosphere instead of air	+2%
9	high water	additional 5.0 eq. water	–2%
10	no water	anhydrous solvents	+-0%
11	high base	2.8 eq. base instead of 2.4 eq.	–5%
12	low base	2.0 eq. base instead of 2.4 eq.	–17%

The electrolysis was performed with 0.25 mmol hydroxamic acid **1a** in a total volume of 10 mL. Active surface of the Pt electrodes was 150 mm² each. Reaction parameters were used as described in general procedure.

5 Experimental section

5.1 General procedure for the synthesis of hydroxamic acids

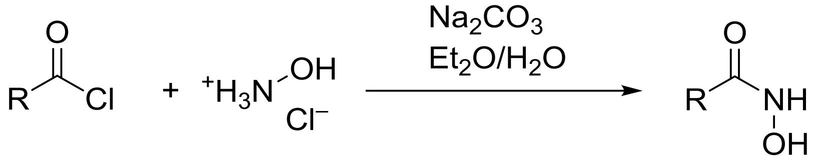

The synthesis was performed according to the literature.[2] To a stirred suspension of hydroxylamine hydrochloride (15 mmol, 1.0 eq.) and sodium carbonate (15 mmol, 1.0 eq.) in 200 mL diethyl ether was added 30 mL water. The mixture was rapidly stirred for 20 min and a solution of the acid chloride (15 mmol, 1.0 eq.) in 10 mL diethyl ether was added in small portions. After 45 min of stirring at rt, the resulted precipitate was filtered and washed with diethyl ether. The product was recrystallized from ethanol, washed with further diethyl ether and dried at 45 °C.

5.2 General procedure for the electrochemical acyl-NDA

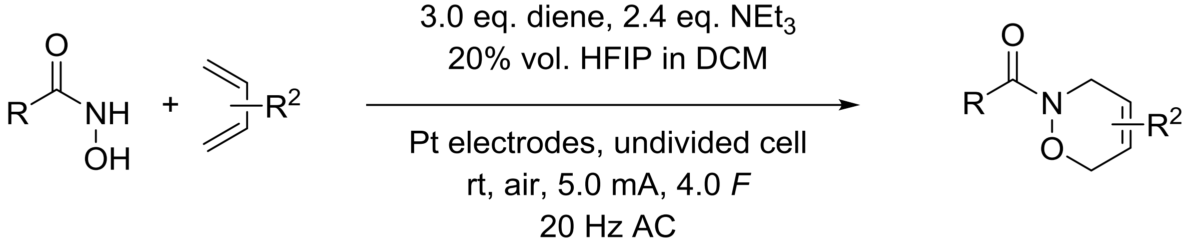

In an undivided cell hydroxamic acid (0.5 mmol, 1.0 eq.) was suspended in 8.0 mL DCM and 2.0 mL HFIP. Under stirring, triethylamine (1.2 mmol, 2.4 eq.) was slowly added and after complete dissolving of the hydroxamic acid, the diene (1.5 mmol, 3.0 eq.) was added. The electrolysis was performed at platinum electrodes (150 mm^2) at 5.0 mA alternating current (20 Hz) until 4.0 *F* were passed through the solution (10 h 43 min). The solvent was removed under reduced pressure to obtain the crude product in high purity. If further purification is needed, silica gel column chromatography (pentane/ethyl acetate) can be performed.

6 Analytical section

6.1 Analytical data of synthesized hydroxamic acids

N-Hydroxy-4-nitrobenzamide (1b)

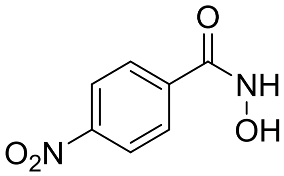

yellow solid, yield: 1279 mg, 47%.

^{1}H NMR (500 MHz, DMSO-d_6): δ = 8.00 (d, ^{3}J = 8.3 Hz, 2H), 8.29 (d, ^{3}J = 8.3 Hz, 2H), 9.31 (br s, 1H), 11.51 (br s, 1H) ppm.
^{13}C NMR (126 MHz, DMSO-d_6): δ = 123.5, 128.3, 138.5, 148.9, 165.1 ppm.
Analytical data are consistent with the literature.[3]

N-Hydroxy-4-(trifluoromethyl)benzamide (1c)

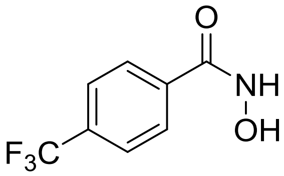

white solid, yield: 2950 mg, 96%.

^{1}H NMR (500 MHz, DMSO-d_6): δ = 7.84 (d, ^{3}J = 7.9 Hz, 2H), 7.96 (d, ^{3}J = 7.9 Hz, 2H), 9.20 (s, 1H), 11.46 (s, 1H) ppm.
^{13}C NMR (126 MHz, DMSO-d_6): δ = 122.8, 125.3, 127.8, 131.2, 136.6, 162.8 ppm.
Analytical data are consistent with the literature.[4]

N-Hydroxy-4-methylbenzamide (1d)

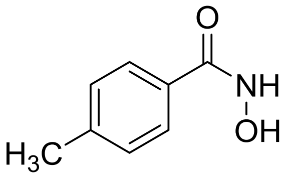

white solid, yield: 1926 mg, 85%.

^{1}H NMR (500 MHz, DMSO-d_6): δ = 2.33 (s, 3H), 7.24 (d, ^{3}J = 8.0 Hz, 2H), 7.65 (d, ^{3}J = 8.0, 2H), 8.91 (s, 1H), 11.11 (s, 1H) ppm.
^{13}C NMR (126 MHz, DMSO-d_6): δ = 20.9, 126.8, 128.8, 129.9, 164.2 ppm.
Analytical data are consistent with the literature.[3]

N-Hydroxy-4-methoxybenzamide (1e)

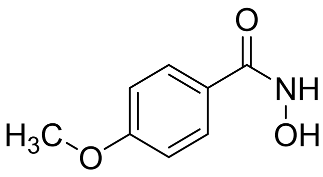

white solid, yield: 2330 mg, 93%.

^{1}H NMR (500 MHz, DMSO-d_6): δ = 3.80 (s, 3H), 6.98 (d, ^{3}J = 8.8 Hz, 2H), 7.74 (d, ^{3}J = 8.8 Hz, 2H), 8.87 (s, 1H), 11.06 (s, 1H) ppm.
^{13}C NMR (126 MHz, DMSO-d_6): δ = 55.3, 113.6, 124.9, 128.6, 161.4, 164.0 ppm.
Analytical data are consistent with the literature.[3]

***N*-Hydroxypivalamide (1h)**

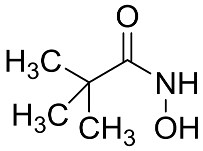

white solid, yield: 1454 mg, 83%.

^{1}H NMR (500 MHz, DMSO-*d*$_6$): *δ* = 1.09 (s, 9H), 8.48 (s, 1H), 10.29 (s, 1H) ppm.
^{13}C NMR (126 MHz, DMSO-*d*$_6$): *δ* = 27.2, 36.9, 174.4 ppm.
Analytical data are consistent with the literature.[4]

***N*-Hydroxy-2-phenylacetamide (1i)**

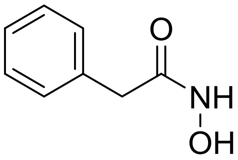

white solid, yield: 1744 mg, 77%.

^{1}H NMR (500 MHz, DMSO-*d*$_6$): *δ* = 3.27 (s, 2H), 7.20–7.30 (m, 5H), 8.88 (br s, 1H), 11.01 (br s, 1H) ppm.
^{13}C NMR (126 MHz, DMSO-*d*$_6$): *δ* = 39.1, 126.5, 128.2, 130.0, 136.3, 167.2 ppm.
Analytical data are consistent with the literature.[4]

6.2 Analytical data of synthesized 1,2-oxazines

(4,5-Dimethyl-3,6-dihydro-2*H*-1,2-oxazin-2-yl)(phenyl)methanone (3a)

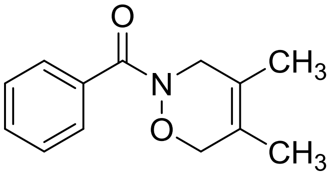

white solid, yield: 91 mg, 85%.

R$_f$ = 0.29 (pentane:ethyl acetate = 9:1).
mp ($CHCl_3$): 72–74 °C.
^{1}H NMR (500 MHz, $CDCl_3$): *δ* = 1.57 (s, 3H), 1.69 (s, 3H), 4.12 (s, 2H), 4.16 (s, 2H), 7.37–7.44 (m, 3H), 7.67–7.69 (m, 2H) ppm.
^{13}C NMR (126 MHz, $CDCl_3$): *δ* = 13.8, 15.4, 46.7, 72.8, 122.8, 128.0, 128.5, 130.9, 133.9, 169.6 ppm.
HR-MS (EI): m/z calcd for $C_{13}H_{15}NO_2^+$ (**3a**) 217.1097, found 217.1101.
IR (ATR): $\tilde{\nu}$ = 2923 (w), 2890 (w), 2847 (w), 1641 (s), 1589 (w), 1578 (w), 1445 (m), 1397 (m), 1361 (m), 1221 (s), 1148 (w), 1000 (m), 979 (w), 891 (w), 796 (m), 764 (w), 718 (m), 707 (s), 696 (s), 677 (m), 620 (w), 564 (w), 513 (w) cm^{-1}.

(4-Methyl-3,6-dihydro-2*H*-1,2-oxazin-2-yl)(phenyl)methanone (3b-d) and **(5-methyl-3,6-dihydro-2*H*-1,2-oxazin-2-yl)(phenyl)methanone (3b-p)**

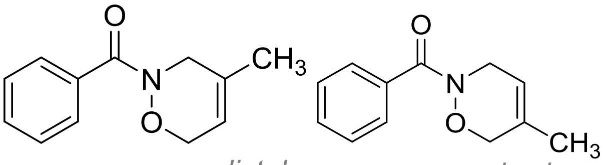

distal *proximal* colourless oil, yield: 67 mg, 66%, *d*:*p* = 77:23.

R_f = 0.21 (pentane:ethyl acetate = 9:1).

^{1}H NMR (500 MHz, $CDCl_3$): δ = 1.66 (s, 0.68H), 1.78 (s, 2.32H), 4.19 (s, 0.46H), 4.22 (s, 1.54H), 4.28 (s, 2H), 5.54 (s, 1H), 7.41 (m, 3H), 7.69 (m, 2H) ppm.

^{13}C NMR (126 MHz, $CDCl_3$): δ = 18.3, 20.0, 43.2, 46.8, 69.7, 72.8, 116.4, 117.6, 128.0, 128.5, 130.3, 130.9, 131.2, 133.9, 169.8 ppm.

HR-MS (EI): m/z calcd for $C_{12}H_{13}NO_2^+$ (**3b**) 203.0941, found 203.0940.

IR (ATR): $\tilde{\nu}$ = 3300 (w), 2847 (w), 1726 (w), 1641 (s), 1601 (m), 1577 (m), 1490 (w), 1447 (m), 1383 (m), 1352 (m), 1224 (m), 1180 (m), 1158 (m), 1136 (m), 1060 (m), 1026 (m), 931 (w), 904 (w), 786 (m), 696 (s), 654 (w), 594 (w) cm^{-1}.

***cis*-(3,6-Dimethyl-3,6-dihydro-2*H*-1,2-oxazin-2-yl)(phenyl)methanone (3c)**

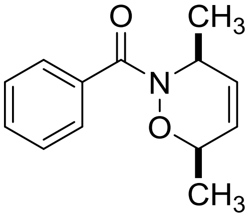

colourless oil, yield: 86 mg, 79%.

R_f = 0.24 (pentane:ethyl acetate = 9:1).

^{1}H NMR (500 MHz, $CDCl_3$): δ = 1.15 (d, ^3J = 6.7 Hz, 3H), 1.40 (d, ^3J = 6.7 Hz, 3H), 4.51 (br s, 1H), 4.90 (br s, 1H), 5.66–5.68 (m, 1H), 5.80–5.83 (m, 1H), 7.37–7.45 (m, 3H), 7.68–7.70 (m, 2H) ppm.

^{13}C NMR (126 MHz, $CDCl_3$): δ = 18.2, 18.8, 48.2, 75.3, 127.9, 128.0, 128.2, 128.4, 130.7, 134.2, 168.5 ppm.

HR-MS (EI): m/z calcd for $C_{13}H_{15}NO_2^+$ (**3c**) 217.1097, found 217.1089.

IR (ATR): $\tilde{\nu}$ = 2977 (w), 2933 (w), 2871 (w), 1661 (m), 1632 (s), 1602 (m), 1577 (m), 1493 (w), 1447 (m), 1406 (s), 1382 (s), 1372 (s), 1309 (m), 1199 (m), 1157 (w), 1130 (w), 1090 (m), 1030 (m), 944 (w), 874 (m), 861 (w), 787 (w), 738 (s), 699 (s), 647 (m), 584 (m), 519 (w) cm^{-1}.

(3-Cyclohexyl-3,6-dihydro-2*H*-1,2-oxazin-2-yl)(phenyl)methanone (3d-d) and **(6-cyclohexyl-3,6-dihydro-2*H*-1,2-oxazin-2-yl)(phenyl)methanone (3d-p)**

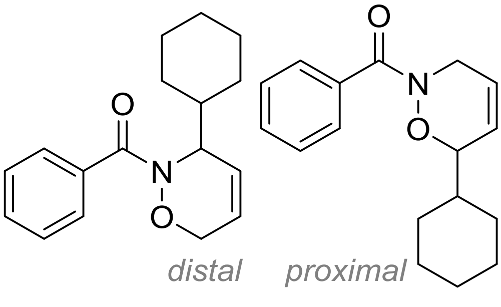

distal *proximal* colourless oil, yield: 116 mg, 86%, *d*:*p* = 35:65.

R_f = 0.18 (pentane:ethyl acetate = 9:1).

^{1}H NMR (500 MHz, $CDCl_3$): δ = 0.82–1.96 (m, 11H), 3.94–4.33 (m, 2H), 4.58–4.88 (m, 1H), 5.78–5.85 (m, 1H), 5.86–5.92 (m, 0.65H), 5.93–6.00 (m, 0.35H), 7.32–7.50 (m, 3H), 7.64–7.74 (m, 2H) ppm.
^{13}C NMR (126 MHz, $CDCl_3$): δ = 26.1, 26.2, 26.3, 26.4, 26.5, 27.8, 28.5, 29.9, 30.0, 41.2, 42.2, 43.1, 56.0, 69.9, 83.3, 123.0, 123.4, 126.9, 127.9, 128.0, 128.7, 128.8, 130.7, 130.9, 134.0, 134.4, 169.5, 170.0 ppm.
HR-MS (EI): m/z calcd for $C_{17}H_{21}NO_2^+$ (**3d**) 271.1567, found 271.1565.
IR (ATR): $\tilde{\nu}$ = 3057 (w), 2924 (m), 2850 (m), 1662 (m), 1643 (s), 1601 (m), 1579 (w), 1494 (w), 1447 (m), 1404 (m), 1379 (m), 1296 (w), 1220 (w), 1073 (w), 1029 (w), 980 (w), 856 (w), 787 (w), 700 (s), 650 (m), 534 (w) cm^{-1}.

Phenyl(3,3,5-trimethyl-3,6-dihydro-2*H*-1,2-oxazin-2-yl)methanone (3e)

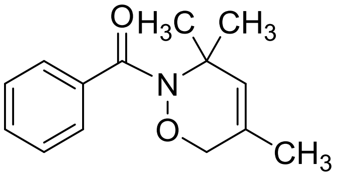

white solid, yield: 42 mg, 36%.
R_f = 0.25 (pentane:ethyl acetate = 9:1).
mp ($CHCl_3$): 76–79 °C.
^{1}H NMR (500 MHz, $CDCl_3$): δ = 1.11 (s, 6H), 1.74 (s, 3H), 4.23 (s, 2H), 5.40 (s, 1H), 7.35–7.44 (m, 3H), 7.70–7.77 (m, 2H) ppm.
^{13}C NMR (126 MHz, $CDCl_3$): δ = 19.9, 26.0, 45.6, 80.2, 127.3, 127.9, 129.2, 130.5, 134.6, 168.6 ppm.
HR-MS (EI): m/z calcd for $C_{14}H_{17}NO_2^+$ (**3e**) 231.1254, found 231.1258.
IR (ATR): $\tilde{\nu}$ = 2971 (w), 2933 (w), 1626 (s), 1579 (w), 1381 (s), 1362 (m), 1312 (m), 1287 (w), 1239 (m), 1216 (m), 1194 (m), 976 (w), 946 (w), 921 (m), 894 (m), 810 (m), 787 (s), 761 (w), 714 (s), 703 (s), 654 (s), 616 (m), 527 (w) cm^{-1}.

***cis*-(7-Oxa-8-azabicyclo[4.2.2]dec-9-en-8-yl)(phenyl)methanone (3f)**

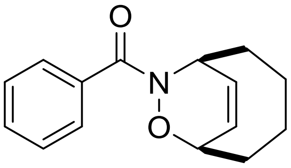

colourless oil, yield: 100 mg, 82%.
R_f = 0.09 (pentane:ethyl acetate = 9:1).
^{1}H NMR (500 MHz, 298 K, $CDCl_3$): δ = 1.60–2.32 (m, 8H), 4.36–4.77 (m, 1H), 5.25 (br s, 1H), 5.91 (br s, 1H), 6.13–6.47 (m, 1H), 7.33–7.45 (m, 3H), 7.55–7.79 (m, 2H) ppm.
^{13}C NMR (126 MHz, 298 K, $CDCl_3$): δ = 22.9, 25.0, 31.4, 34.6, 51.0, 77.3, 127.1, 127.8, 128.6, 130.3, 130.9, 134.9, 167.9 ppm.
^{1}H NMR (500 MHz, 248 K, $CDCl_3$): δ = 1.40–2.38 (m, 8H), 4.40–4.45 (m, 0.33H), 4.74 (s, 0.66H), 5.22–5.28 (m, 1H), 5.82 (dd, ^3J = 4.4, 10.0 Hz, 0.33H), 5.94 (br s, 0.66H), 6.16 (dd, 7.0, 10.0 Hz, 0.33H), 6.32 (br s, 0.66H), 7.36–7.47 (m, 3H), 7.48–7.52 (m, 0.66H), 7.62–7.66 (m, 1.33H) ppm.
^{13}C NMR (126 MHz, 248 K, $CDCl_3$): δ = 21.7, 22.5, 24.5, 25.7, 31.0, 31.7, 34.2, 34.5, 50.6, 55.8, 77.7, 126.3, 127.4, 127.8, 128.2, 128.7, 130.0, 130.3, 130.9, 134.2, 134.3, 167.2, 170.4 ppm.
HR-MS (EI): m/z calcd for $C_{15}H_{17}NO_2^+$ (**3f**) 243.1254, found 243.1253.

IR (ATR): $\tilde{\nu}$ = 3053 (w), 2916 (m), 2856 (w), 1664 (m), 1617 (s), 1574 (m), 1447 (m), 1424 (m), 1381 (m), 1315 (m), 1287 (m), 1245 (m), 1212 (m), 1182 (s), 1096 (w), 1054 (m), 987 (m), 872 (w), 784 (s), 697 (s), 654 (s), 544 (w) cm^{-1}.

(3-Phenethyl-3,6-dihydro-2*H*-1,2-oxazin-2-yl)(phenyl)methanone (3h-d) and **(6-phenethyl-3,6-dihydro-2*H*-1,2-oxazin-2-yl)(phenyl)methanone (3h-p)**

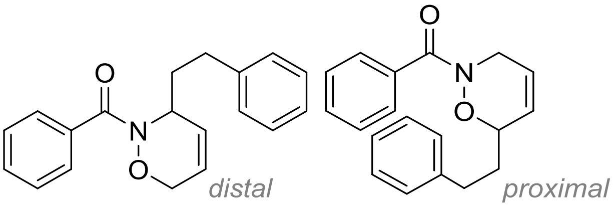

colourless oil, yield: 98 mg, 67% *d*:*p* = 50:50.

R$_f$ = 0.18 (pentane:ethyl acetate = 9:1).
^{1}H NMR (500 MHz, $CDCl_3$): δ = 1.59–1.81 (m, 1H), 1.94–2.05 (m, 0.57H), 2.08–2.19 (m, 0.57H), 2.23–2.34 (m, 0.43H), 2.42–2.52 (m, 0.43H), 2.69–2.74 (m, 1H), 3.99–4.11 (m, 1H), 4.20–4.35 (m, 1H), 4.53–4.63 (m, 0.43H), 4.89 (s, 0.57) 6.80–6.88 (m, 1H), 7.03–7.44 (m, 7H), 7.58–7.68 (m, 2H) ppm.
^{13}C NMR (126 MHz, $CDCl_3$): δ = 31.2, 32.5, 34.8, 35.1, 43.0, 52.0, 69.8, 78.6, 122.8, 123.4, 126.1, 126.1, 126.9, 128.0, 128.0, 128.4, 128.5, 128.5, 128.8, 130.8, 131.0, 134.1, 134.1, 141.2, 141.7, 169.5, 169.7 ppm.
HR-MS (EI): m/z calcd for $C_{19}H_{19}NO_2^+$ (**3h**) 293.1410, found 293.1405.
IR (ATR): $\tilde{\nu}$ = 2924 (w), 2847 (w), 1661 (m), 1633 (s), 1602 (m), 1577 (m), 1496 (m), 1447 (m), 1408 (w), 1376 (w), 1236 (m), 1200 (w), 1155 (w), 1041 (m), 1025 (m), 914 (w), 869 (w), 785 (w), 750 (m), 698 (s), 631 (w), 535 (w) cm^{-1}.

***cis*-(3-(Hydroxymethyl)-6-methyl-3,6-dihydro-2*H*-1,2-oxazin-2-yl)(phenyl)methanone (3k)**

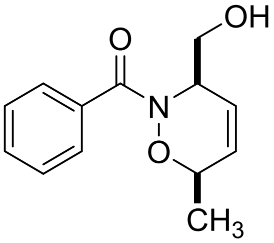
colourless oil, yield: 79 mg, 68%.

R$_f$ = 0.08 (pentane:ethyl acetate = 7:3).
^{1}H NMR (500 MHz, $CDCl_3$): δ = 1.39 (d, ^3J = 6.7 Hz, 3H), 2.50 (s, 1H), 3.55 – 3.66 (m, 2H), 4.58 (s, 1H), 4.85 (br s, 1H), 5.67–5.73 (m, 1H), 5.90–5.97 (m, 1H), 7.34–7.48 (m, 3H), 7.64–7.70 (m, 2H) ppm.
^{13}C NMR (126 MHz, $CDCl_3$): δ = 18.3, 49.3, 63.6, 80.3, 123.5, 128.2, 128.4, 130.3, 131.1, 134.0, 169.0 ppm.
HR-MS (EI): m/z calcd for $C_{13}H_{15}NO_3^+$ (**3k**) 233.1046, found 233.1048.
IR (ATR): $\tilde{\nu}$ = 3410 (m), 3048 (w), 2977 (w), 2931 (w), 2870 (w), 1614 (s), 1574 (m), 1494 (w), 1447 (s), 1412 (s), 1315 (w), 1199 (m), 1145 (w), 1048 (s), 1002 (m), 984 (w), 920 (w), 871 (m), 844 (m), 787 (w), 735 (m), 700 (s), 647 (w), 536 (w) cm^{-1}.

(4,5-Dimethyl-3,6-dihydro-2*H*-1,2-oxazin-2-yl)(4-(trifluoromethyl)phenyl)methanone (5a)

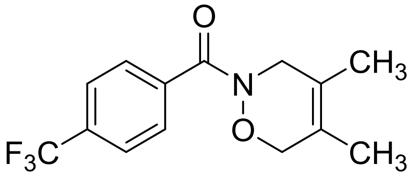
white solid, yield: 127 mg, 89%.

R$_f$ = 0.33 (pentane:ethyl acetate = 9:1).
mp ($CHCl_3$): 112–115 °C.
^{1}H NMR (500 MHz, $CDCl_3$): δ = 1.57 (s, 3H), 1.71 (s, 3H), 4.09 (s, 2H), 4.18 (s, 2H), 7.68 (d, ^3J = 8.2 Hz, 2H), 7.78 (d, ^3J = 8.2 Hz, 2H) ppm.
^{13}C NMR (126 MHz, $CDCl_3$): δ = 13.8, 15.4, 46.3, 73.1, 120.6, 121.9, 122.7, 122.8, 125.0, 125.1, 127.1, 129.0, 132.2, 134.4, 132.7, 132.9, 137.3, 168.0 ppm.
HR-MS (EI): m/z calcd for $C_{14}H_{14}F_3NO_2^+$ (**5a**) 285.0971, found 285.0973.
IR (ATR): $\tilde{\nu}$ = 2937 (w), 2833 (w), 1633 (m), 1579 (w), 1510 (w), 1422 (m), 1390 (w), 1320 (s), 1234 (m), 1160 (m), 1112 (s), 1064 (s), 1021 (m), 1012 (m), 975 (m), 891 (w), 853 (s), 762 (m), 747 (m), 700 (m), 624 (w), 511 (w) cm^{-1}.

(4,5-Dimethyl-3,6-dihydro-2*H*-1,2-oxazin-2-yl)(*p*-tolyl)methanone (6a)

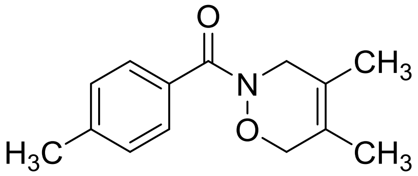
white solid, yield: 94 mg, 81%.

R$_f$ = 0.15 (pentane:ethyl acetate = 9:1).
mp ($CHCl_3$): 84–88 °C.
^{1}H NMR (500 MHz, $CDCl_3$): δ = 1.57 (s, 3H), 1.69 (s, 3H), 2.37 (s, 3H), 4.07–4.21 (m, 4H), 7.19 (d, ^3J = 8.1 Hz, 2H), 7.61 (d, ^3J = 8.1 Hz, 2H) ppm.
^{13}C NMR (126 MHz, $CDCl_3$): δ = 13.8, 15.5, 21.5, 72.7, 122.0, 122.8, 128.7, 128.7, 130.9, 141.3, 169.7 ppm.
HR-MS (EI): m/z calcd for $C_{14}H_{17}NO_2^+$ (**6a**) 231.1254, found 231.1257.
IR (ATR): $\tilde{\nu}$ = 2986 (w), 2924 (w), 2840 (w), 1637 (s), 1611 (m), 1571 (w), 1434 (m), 1409 (m), 1360 (m), 1227 (s), 1180 (w), 1167 (w), 1152 (w), 1027 (s) 1017 (m), 980 (w), 892 (w), 828 (s), 800 (w), 748 (m), 740 (s), 634 (w), 564 (w), 513 (w) cm^{-1}.

(4,5-Dimethyl-3,6-dihydro-2*H*-1,2-oxazin-2-yl)(4-methoxyphenyl)methanone (7a)

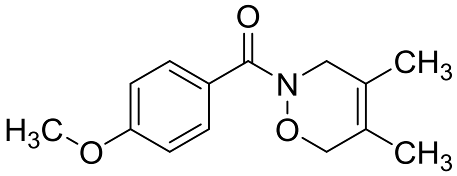
white solid, yield: 95 mg, 77%.

R$_f$ = 0.17 (pentane:ethyl acetate = 9:1).
mp ($CHCl_3$): 103–107 °C.
^{1}H NMR (500 MHz, $CDCl_3$): δ = 1.56 (s, 3H), 1.69 (s, 3H), 3.81 (s, 3H), 4.07–4.22 (m, 4H), 6.88 (d, ^3J = 8.8 Hz, 2H), 7.72 (d, ^3J = 8.8 Hz, 2H) ppm.
^{13}C NMR (126 MHz, $CDCl_3$): δ = 13.8, 15.5, 46.8, 55.4, 72.7, 113.3, 122.1, 122.7, 125.8, 130.9, 161.9, 169.3 ppm.
HR-MS (EI): m/z calcd for $C_{14}H_{17}NO_3^+$ (**7a**) 247.1203, found 247.1210.

IR (ATR): $\tilde{\nu}$ = 2934 (w), 2839 (w), 1739 (w), 1627 (s), 1605 (m), 1579 (m), 1508 (m), 1457 (w), 1440 (w), 1415 (m), 1392 (m), 1358 (m), 1302 (m), 1254 (s), 1220 (m), 1181 (m), 1164 (m), 1111 (w), 1025 (s), 982 (w), 947 (w), 895 (w), 845 (s), 801 (m), 760 (m), 717 (w), 657 (w), 625 (w), 584 (w), 566 (w), 505 (w) cm^{-1}.

1-(4,5-Dimethyl-3,6-dihydro-2*H*-1,2-oxazin-2-yl)ethan-1-one (8a)

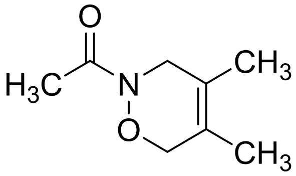

colourless oil, yield: 50 mg, 64%.

R$_f$ = 0.25 (pentane:ethyl acetate = 7:3).
^{1}H NMR (500 MHz, CDCl$_3$): δ = 1.58 (s, 3H), 1.66 (s, 3H), 2.11 (s, 3H), 4.01 (s, 2H), 4.20 (s, 2H) ppm.
^{13}C NMR (126 MHz, CDCl$_3$): δ = 14.0, 15.5, 20.0, 45.1, 73.0, 122.3, 122.7, 169.7 ppm.
HR-MS (EI): m/z calcd for $C_8H_{13}NO_2^+$ (**8a**) 155.0941, found 155.0939.
IR (ATR): $\tilde{\nu}$ = 2984 (w), 2918 (w), 2841 (w), 1659 (s), 14402 (s), 1408 (s), 1361 (m), 1222 (s), 1177 (w), 1151 (w), 1034 (w), 1010 (w), 971 (m), 924 (w), 901 (w), 780 (w), 658 (m), 581 (w) cm^{-1}.

1-(4,5-Dimethyl-3,6-dihydro-2*H*-1,2-oxazin-2-yl)octan-1-one (9a)

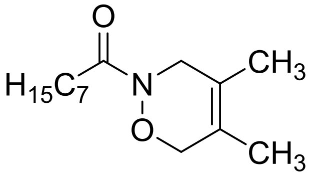

colourless oil, yield: 55 mg, 46%.

R$_f$ = 0.42 (pentane:ethyl acetate = 9:1).
^{1}H NMR (500 MHz, CDCl$_3$): δ = 0.87 (t, ^3J = 6.7, 3H), 1.23–1.36 (m, 8H), 1.59 (s, 3H), 1.61–1.65 (m, 2H), 1.67 (s, 3H), 2.38–2.46 (m, 2H), 4.02 (s, 2H), 4.20 (s, 2H) ppm.
^{13}C NMR (126 MHz, CDCl$_3$): δ = 13.9, 14.2, 15.5, 22.8, 24.9, 29.2, 29.6, 31.9, 32.3, 45.2, 73.1, 122.3, 122.6, 172.6 ppm.
HR-MS (EI): m/z calcd for $C_{14}H_{25}NO_2^+$ (**10a**) 239.1880, found 239.1891.
IR (ATR): $\tilde{\nu}$ = 2954 (m), 2926 (s), 2856 (m), 1737 (m), 1728 (m), 1660 (s), 1440 (m), 1413 (m), 1386 (m), 1217 (m), 1152 (w), 1051 (w), 1015 (w), 724 (w) cm^{-1}.

1-(4,5-Dimethyl-3,6-dihydro-2*H*-1,2-oxazin-2-yl)-2,2-dimethylpropan-1-one (10a)

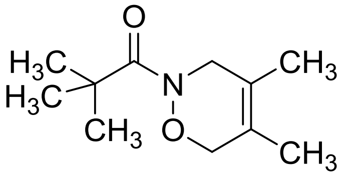

colourless oil, yield: 53 mg, 54%.

R$_f$ = 0.58 (pentane:ethyl acetate = 9:1).
^{1}H NMR (500 MHz, CDCl$_3$): δ = 1.27 (s, 9H), 1.61 (s, 3H), 1.68 (s, 3H), 3.98 (s, 2H), 4.21 (s, 2H) ppm.
^{13}C NMR (126 MHz, CDCl$_3$): δ = 13.9, 15.5, 26.9, 39.3, 47.1, 72.8, 122.1, 122.6, 177.5 ppm.
HR-MS (EI): m/z calcd for $C_{11}H_{19}NO_2^+$ (**10a**) 197.1410, found 197.1415.

IR (ATR): $\tilde{\nu}$ = 2953 (w), 2909 (w), 2160 (w), 1633 (s), 1481 (m), 1461 (w), 1445 (m), 1406 (s), 1382 (m), 1360 (s), 1250 8w), 1202 (s), 1170 (w), 1152 (w), 1045 (w), 1014 (m), 982 (m), 954 (w), 902 (w), 827 (w), 827 (m), 558 (m) cm^{-1}.

1-(4,5-Dimethyl-3,6-dihydro-2*H*-1,2-oxazin-2-yl)-2-phenylethan-1-one (11a)

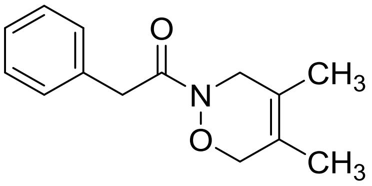

brownish oil, yield: 47 mg, 41%.

R$_f$ = 0.23 (pentane:ethyl acetate = 9:1).
^{1}H NMR (500 MHz, $CDCl_3$): δ = 1.55 (s, 3H), 1.66 (s, 3H), 3.79 (s, 2H), 4.04 (s, 4H), 7.21–7.26 (m, 1H), 7.28–7.34 (m, 4H) ppm.
^{13}C NMR (126 MHz, $CDCl_3$): δ = 13.9, 15.4, 39.9, 45.4, 73.1, 122.0, 122.7, 126.9, 128.6, 129.4, 135.2, 170.1 ppm.
HR-MS (EI): m/z calcd for $C_{14}H_{17}NO_2^+$ (**11a**) 231.1254, found 231.1251.
IR (ATR): $\tilde{\nu}$ = 3030 (w), 2916 (w), 2842 (w), 1652 (s), 1602 (w), 1496 (m), 1454 (s), 1421 (s), 1360 (m), 1247 (w), 1222 (m), 1182 (m), 1151 (w), 959 (w), 900 (w), 797 (w), 746 (m), 710 (s), 694 (m), 650 (w), 582 (w), 516 (w) cm^{-1}.

***tert*-Butyl 4,5-dimethyl-3,6-dihydro-2*H*-1,2-oxazine-2-carboxylate (12a)**

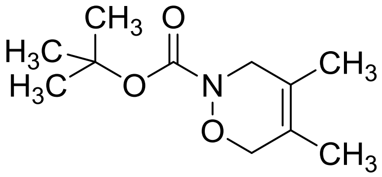

colourless oil, yield: 80 mg, 75%.

R$_f$ = 0.31 (pentane:ethyl acetate = 9:1).
^{1}H NMR (500 MHz, $CDCl_3$): δ = 1.49 (s, 9H), 1.58 (s, 3H), 1.65 (s, 3H), 3.88 (s, 2H), 4.18 (s, 2H) ppm.
^{13}C NMR (126 MHz, $CDCl_3$): δ = 13.9, 15.4, 28.5, 48.7, 71.4, 81.5, 122.2, 123.3, 155.1 ppm.
HR-MS (EI): m/z calcd for $C_{11}H_{19}NO_3^+$ (**12a**) 213.1359, found 213.1359.
IR (ATR): $\tilde{\nu}$ = 2977 (m), 2923 (m), 2852 (m), 1705 (s), 1477 (w), 1456 (w), 1390 (m), 1367 (s), 122 (m), 1239 (m), 1227 (m), 1170 (s), 1137 (s), 1087 (s), 1010 (m), 899 (w), 863 (m), 796 (m), 523 (w) cm^{-1}.

Benzyl 4,5-dimethyl-3,6-dihydro-2*H*-1,2-oxazine-2-carboxylate (13a)

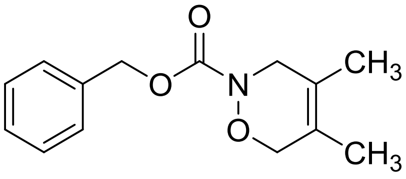

colourless oil, yield: 119 mg, 96%.

R$_f$ = 0.26 (pentane:ethyl acetate = 9:1).
^{1}H NMR (500 MHz, $CDCl_3$): δ = 1.57 (s, 3H), 1.65 (s, 3H), 3.96 (s, 2H), 4.21 (s, 2H), 5.21 (s, 2H), 7.24 – 7.44 (m, 4H) ppm.
^{13}C NMR (126 MHz, $CDCl_3$): δ = 13.8, 15.2, 48.5, 67.6, 71.6, 121.8, 123.2, 128.1, 128.2, 128.5, 136.2, 155.6 ppm.

HR-MS (EI): m/z calcd for $C_{14}H_{17}NO_3^+$ (**13a**) 247.1203, found 247.1199.
IR (ATR): $\tilde{\nu}$ = 2848 (w), 1709 (s), 1499 (w), 1409 (m), 1339 (m), 1250 (w), 1216 (s), 1139 (m), 1029 (m), 1009 (w), 976 (w), 910 (w), 814 (w), 751 (m), 697 (s), 606 (w), 549 (w) cm^{-1}.

6.3 NMR-Spectra of the synthesized 1,2-oxazines

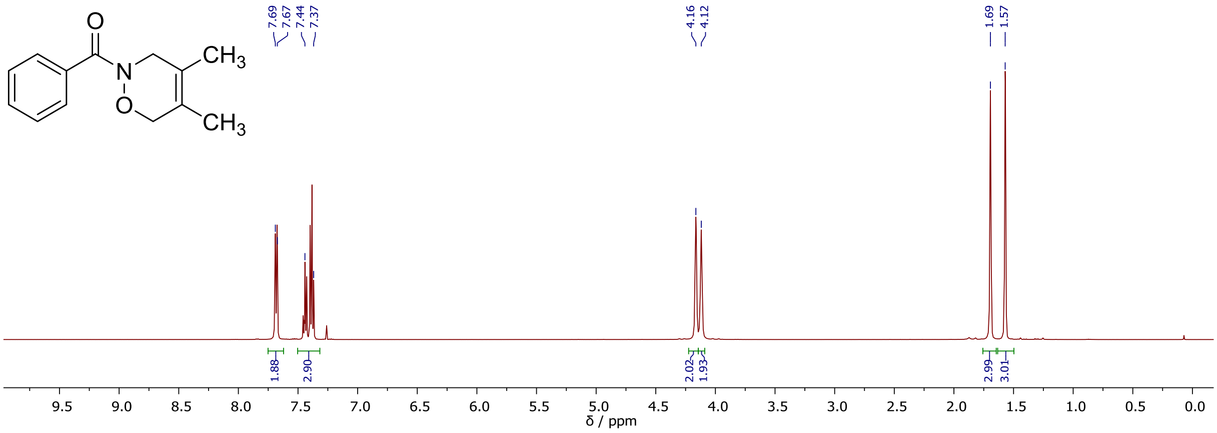

Figure S2: ^{1}H NMR spectrum of (4,5-dimethyl-3,6-dihydro-2*H*-1,2-oxazin-2-yl)(phenyl)methanone (**3a**) in $CDCl_3$ at 500 MHz and rt.

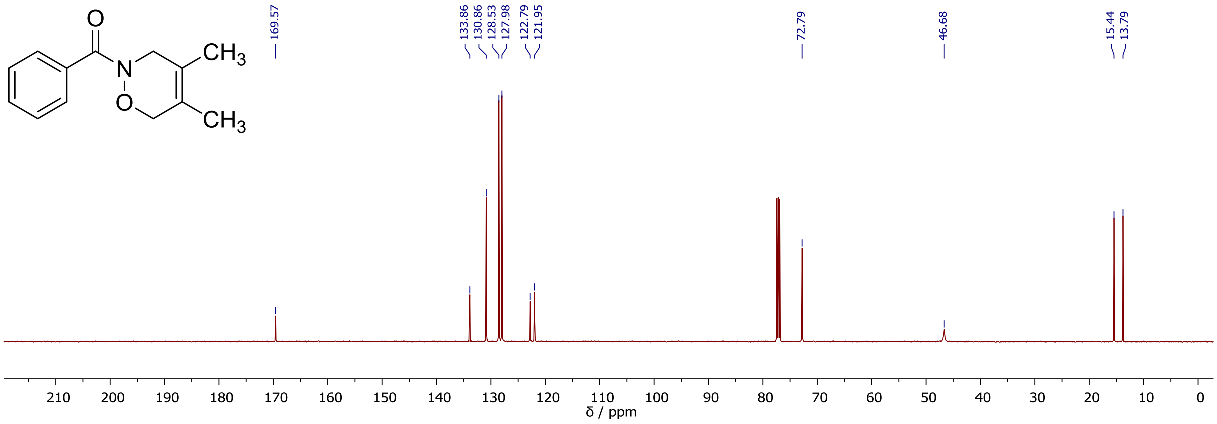

Figure S3: ^{13}C NMR spectrum of (4,5-dimethyl-3,6-dihydro-2*H*-1,2-oxazin-2-yl)(phenyl)methanone (**3a**) in $CDCl_3$ at 126 MHz and rt.

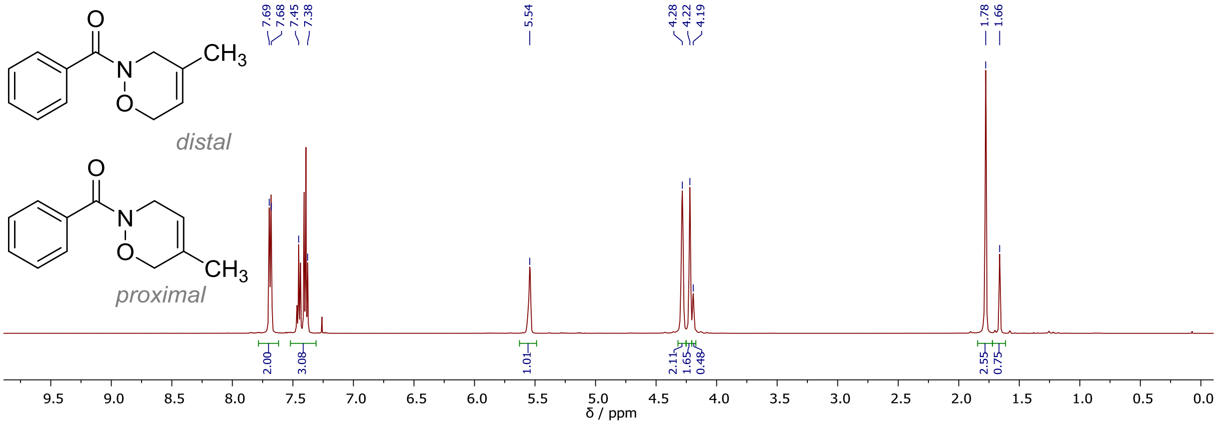

Figure S4: ^{1}H NMR spectrum of (4-methyl-3,6-dihydro-2*H*-1,2-oxazin-2-yl)(phenyl)methanone (**3b-d**) and (5-methyl-3,6-dihydro-2*H*-1,2-oxazin-2-yl)(phenyl)methanone (**3b-p**) in $CDCl_3$ at 500 MHz and rt.

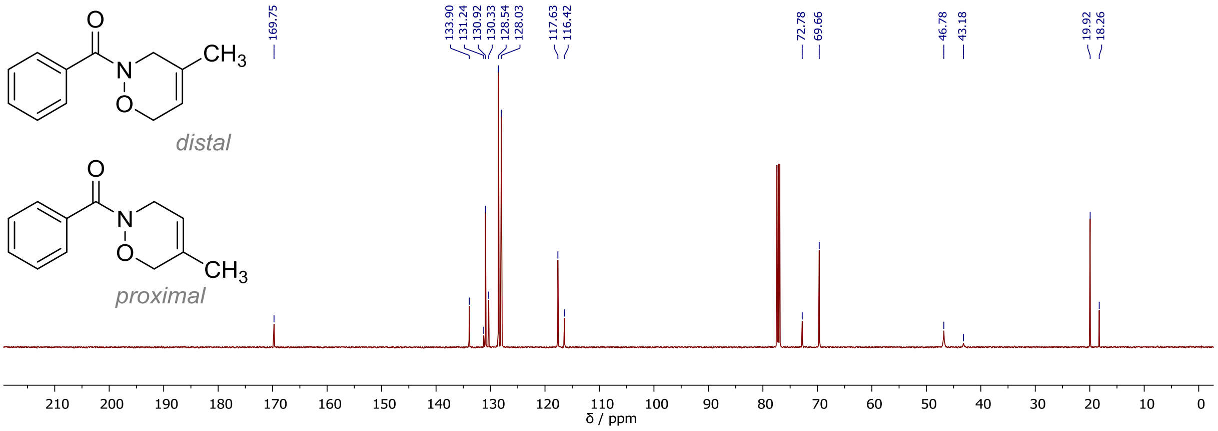

Figure S5: ^{13}C NMR spectrum of (4-methyl-3,6-dihydro-2*H*-1,2-oxazin-2-yl)(phenyl)methanone (**3b-d**) and (5-methyl-3,6-dihydro-2*H*-1,2-oxazin-2-yl)(phenyl)methanone (**3b-p**) in $CDCl_3$ at 126 MHz and rt.

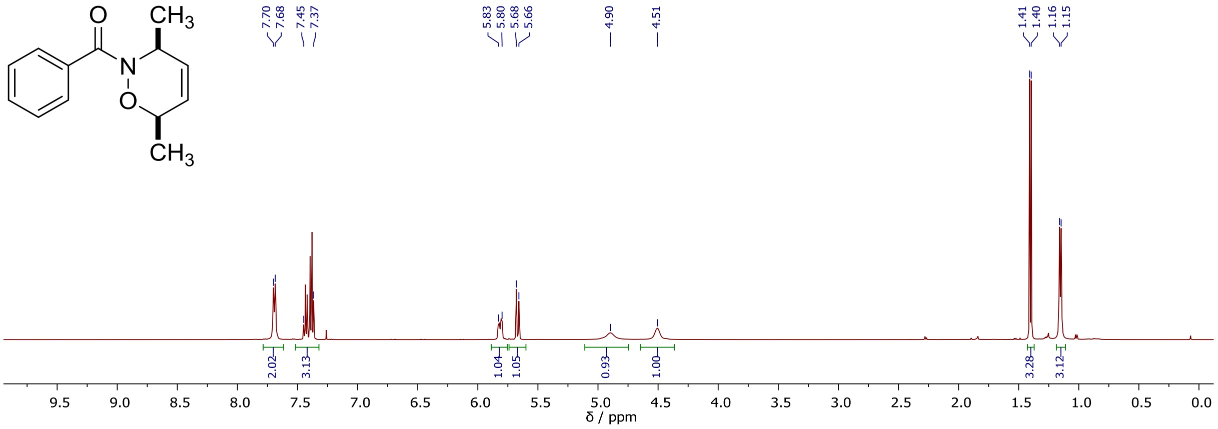

Figure S6: ^{1}H NMR spectrum of *cis*-(3,6-dimethyl-3,6-dihydro-2*H*-1,2-oxazin-2-yl)(phenyl)methanone (**3c**) in $CDCl_3$ at 500 MHz and rt.

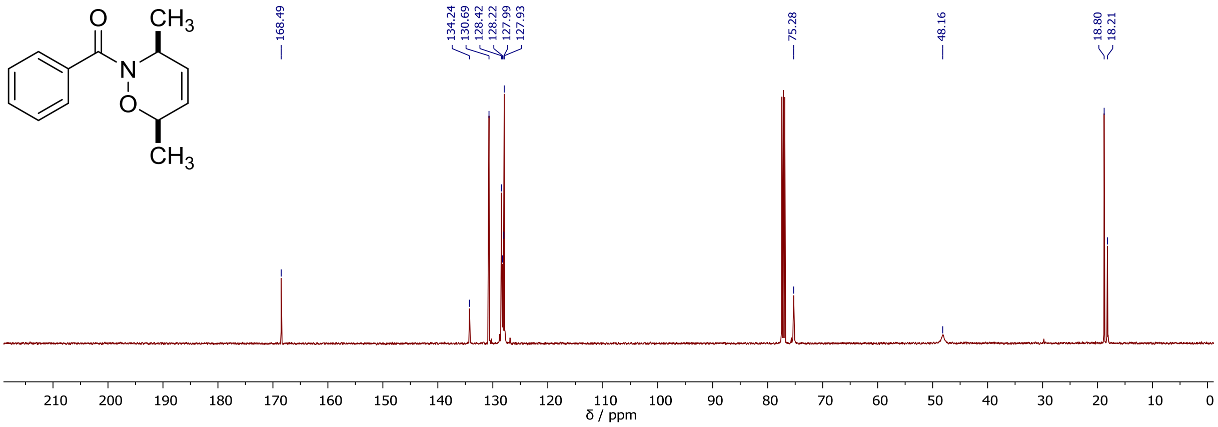

Figure S7: ^{13}C NMR spectrum of *cis*-(3,6-dimethyl-3,6-dihydro-2*H*-1,2-oxazin-2-yl)(phenyl)methanone (**3c**) in $CDCl_3$ at 126 MHz and rt.

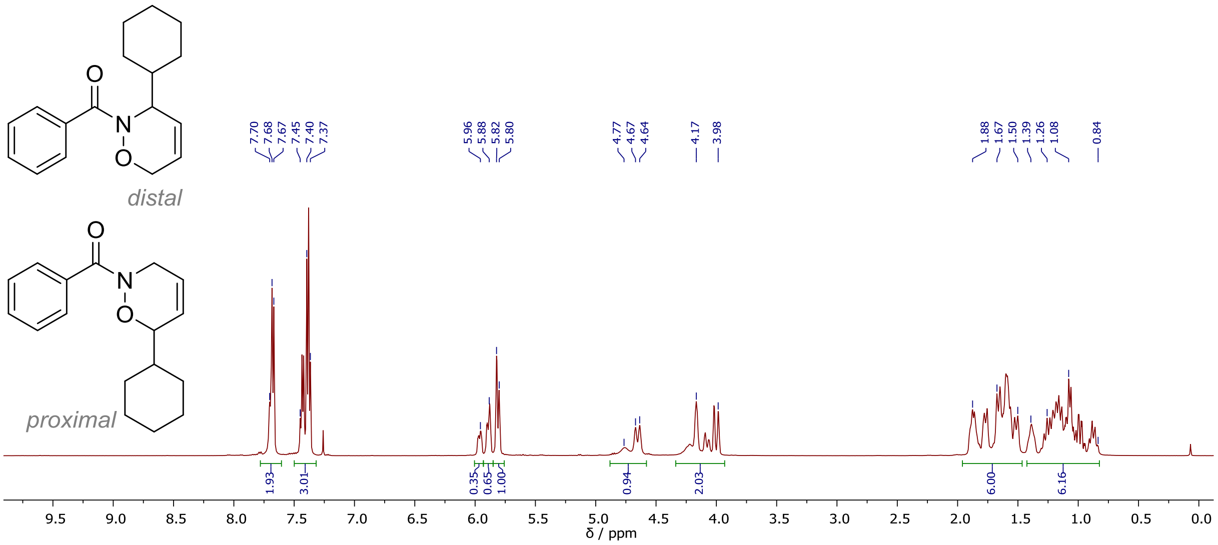

Figure S 8: ^{1}H NMR spectrum of (3-cyclohexyl-3,6-dihydro-2*H*-1,2-oxazin-2-yl)(phenyl)methanone (**3d-d**) and (6-cyclohexyl-3,6-dihydro-2*H*-1,2-oxazin-2-yl)(phenyl)methanone (**3d-p**) in $CDCl_3$ at 500 MHz and rt.

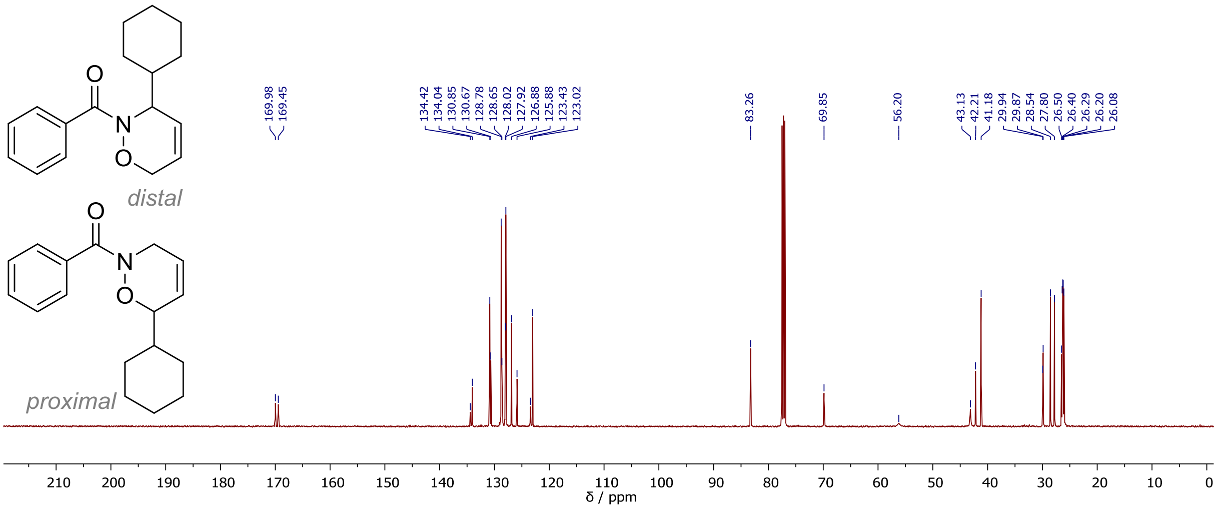

Figure S9: ^{13}C NMR spectrum of (3-cyclohexyl-3,6-dihydro-2*H*-1,2-oxazin-2-yl)(phenyl)methanone (**3d-d**) and (6-cyclohexyl-3,6-dihydro-2*H*-1,2-oxazin-2-yl)(phenyl)methanone (**3d-p**) in $CDCl_3$ at 126 MHz and rt.

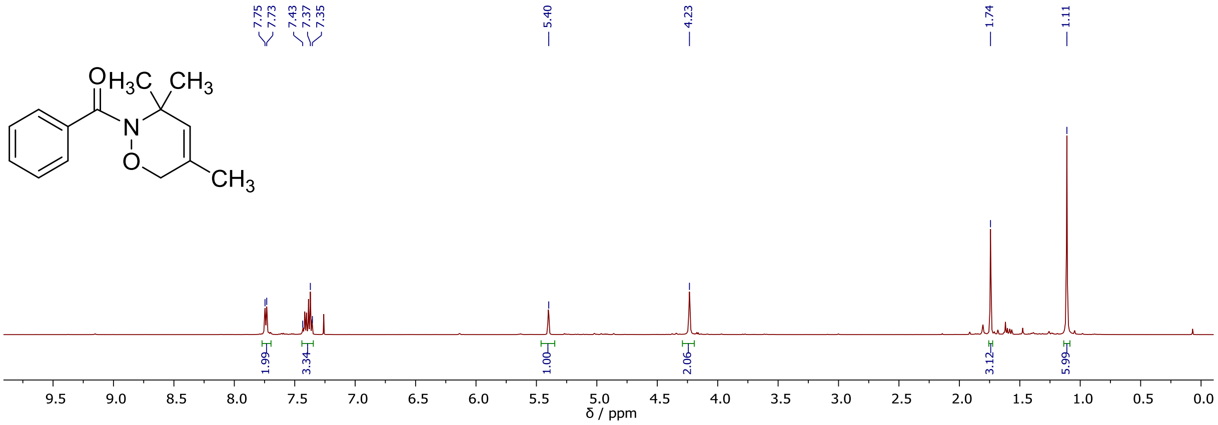

Figure S10: ^{1}H NMR spectrum of phenyl(3,3,5-trimethyl-3,6-dihydro-2*H*-1,2-oxazin-2-yl)methanone (**3e**) in $CDCl_3$ at 500 MHz and rt.

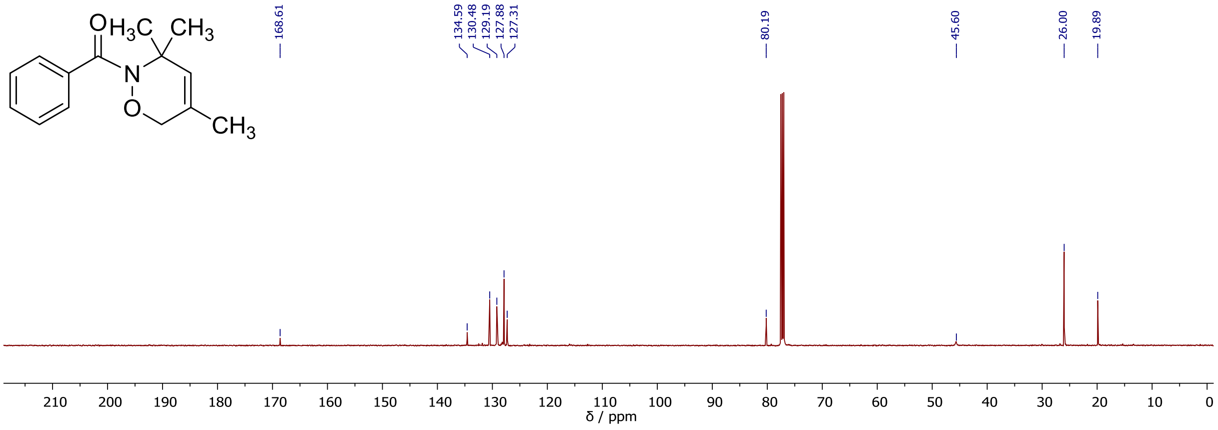

Figure S11: ^{13}C NMR spectrum of phenyl(3,3,5-trimethyl-3,6-dihydro-2*H*-1,2-oxazin-2-yl)methanone (**3e**) in $CDCl_3$ at 126 MHz and rt.

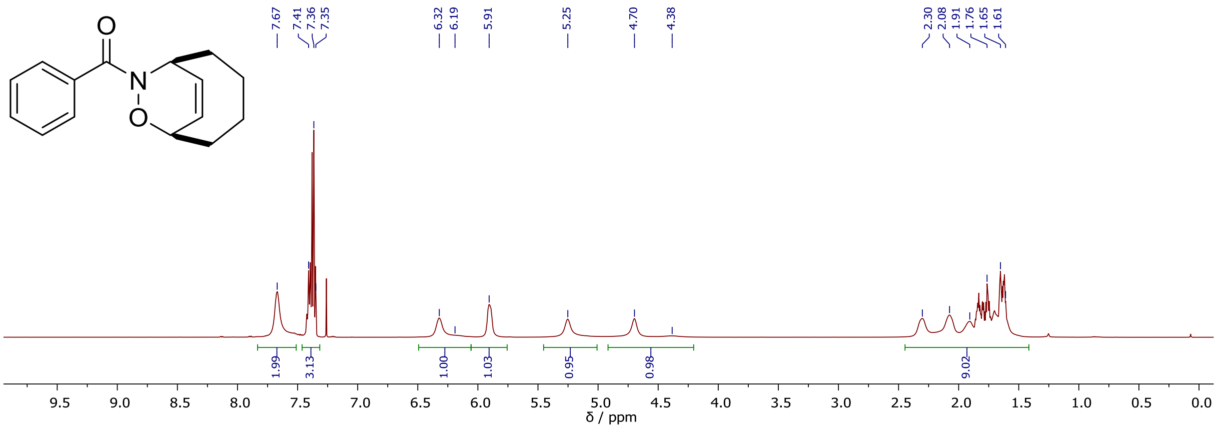

Figure S12: ^{1}H NMR spectrum of *cis*-(7-oxa-8-azabicyclo[4.2.2]dec-9-en-8-yl)(phenyl)methanone (**3f**) in $CDCl_3$ at 500 MHz and rt.

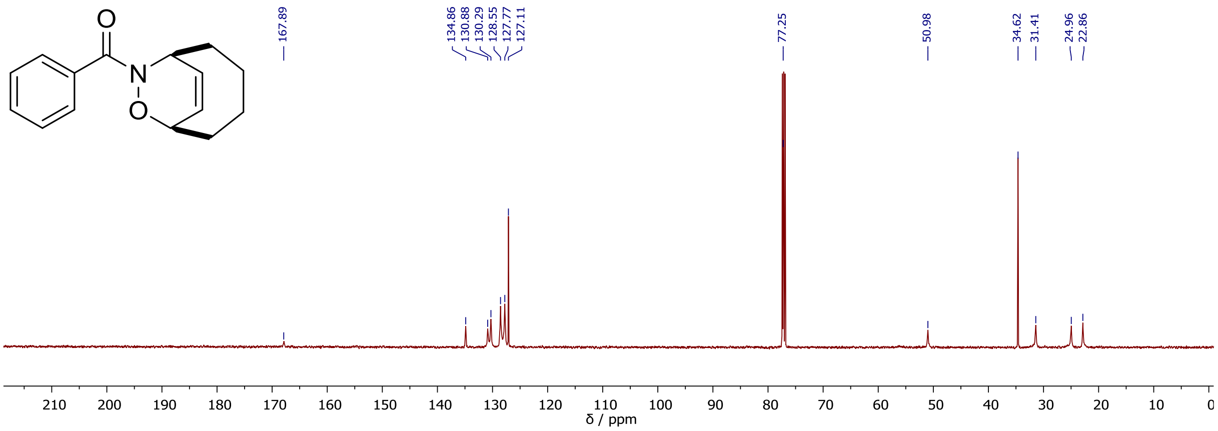

Figure S13: ^{13}C NMR spectrum of *cis*-(7-oxa-8-azabicyclo[4.2.2]dec-9-en-8-yl)(phenyl)methanone (**3f**) in $CDCl_3$ at 126 MHz and rt.

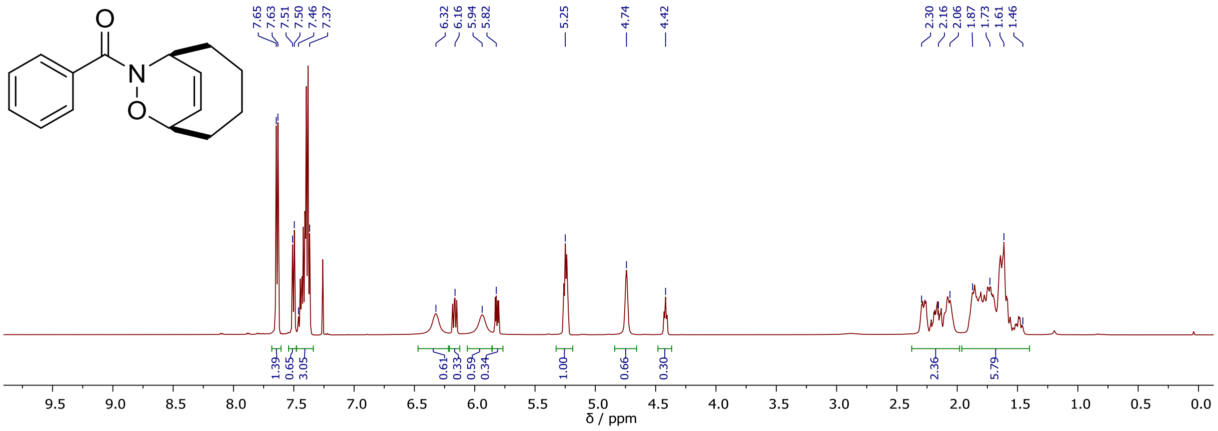

Figure S14: ^{1}H NMR spectrum of *cis*-(7-oxa-8-azabicyclo[4.2.2]dec-9-en-8-yl)(phenyl)methanone (**3f**) in $CDCl_3$ at 500 MHz and 248 K.

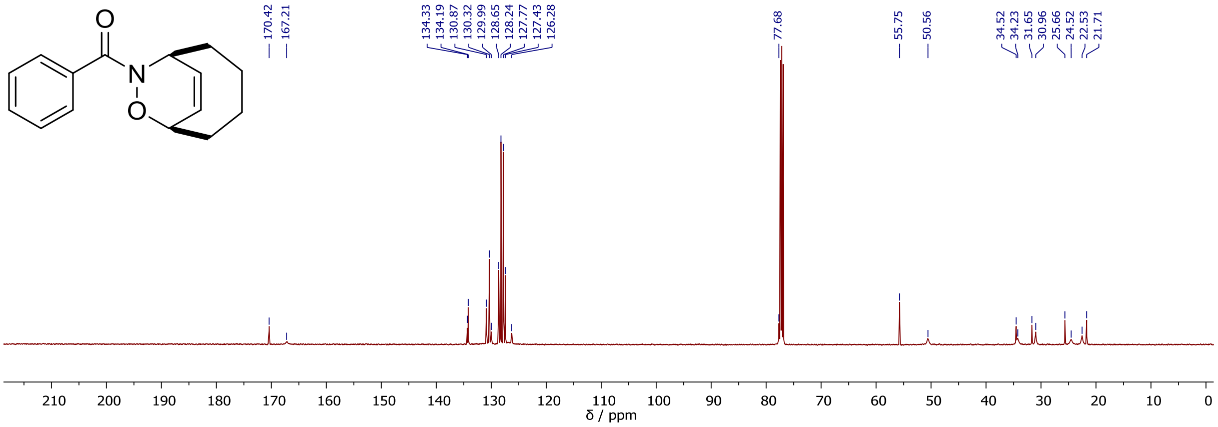

Figure S15: ^{13}C NMR spectrum of *cis*-(7-oxa-8-azabicyclo[4.2.2]dec-9-en-8-yl)(phenyl)methanone (**3f**) in $CDCl_3$ at 126 MHz and 248 K.

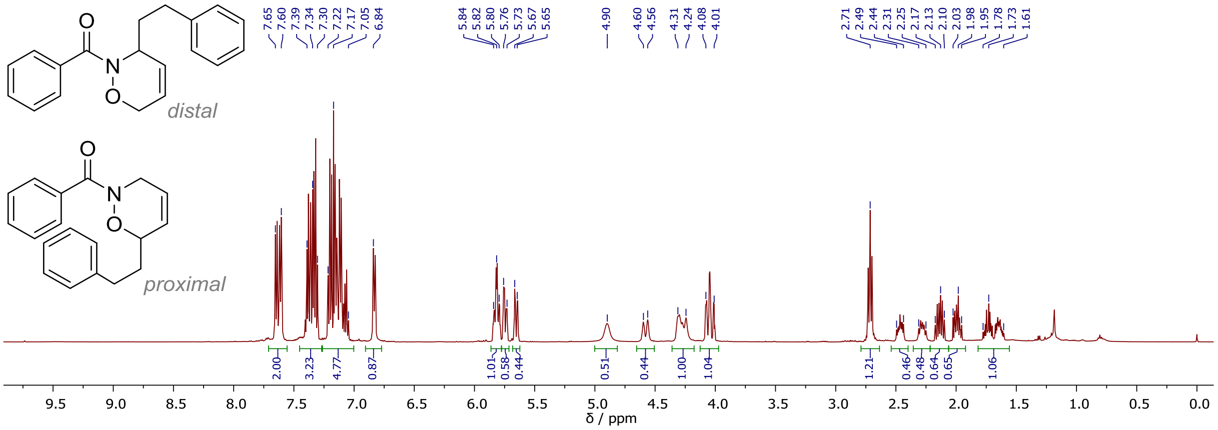

Figure S16: ^{1}H NMR spectrum of (3-phenethyl-3,6-dihydro-2*H*-1,2-oxazin-2-yl)(phenyl)methanone (**3h-d**) and (6-phenethyl-3,6-dihydro-2*H*-1,2-oxazin-2-yl)(phenyl)methanone (**3h-p**) in $CDCl_3$ at 500 MHz and rt.

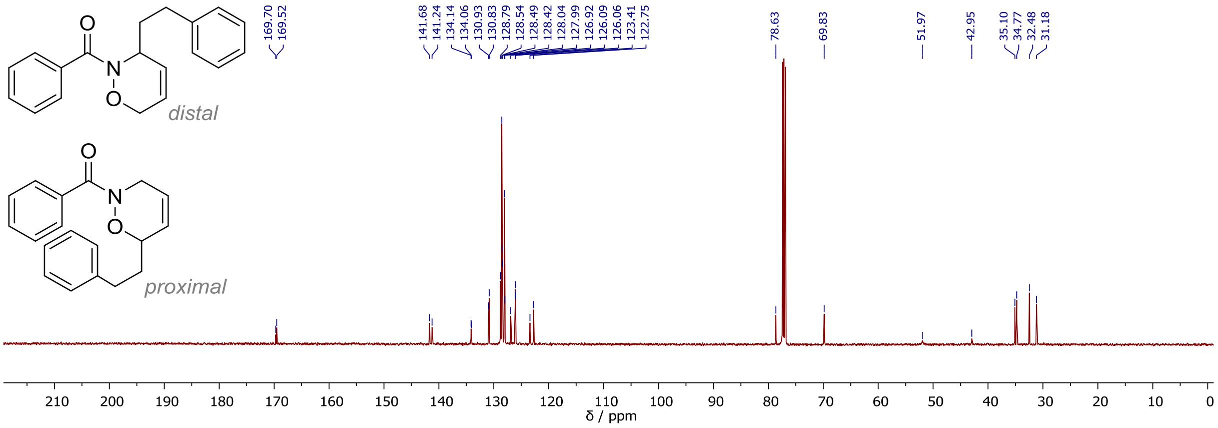

Figure S17: ^{13}C NMR spectrum of (3-phenethyl-3,6-dihydro-2*H*-1,2-oxazin-2-yl)(phenyl)methanone (**3h-d**) and (6-phenethyl-3,6-dihydro-2*H*-1,2-oxazin-2-yl)(phenyl)methanone (**3h-p**) in $CDCl_3$ at 126 MHz and rt.

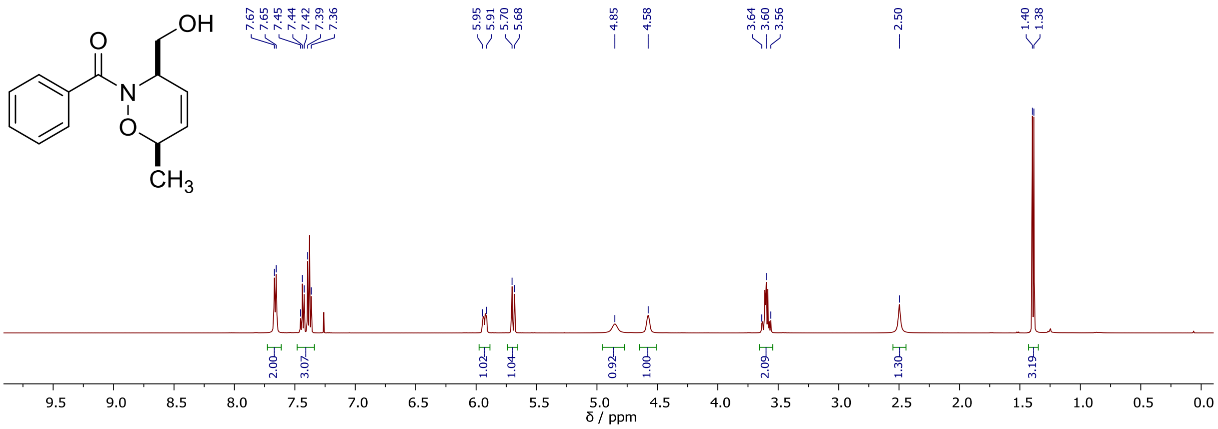

Figure S18: ^{1}H NMR spectrum of *cis*-(3-(hydroxymethyl)-6-methyl-3,6-dihydro-2*H*-1,2-oxazin-2-yl)(phenyl)methanone (**3k**) in $CDCl_3$ at 500 MHz and rt.

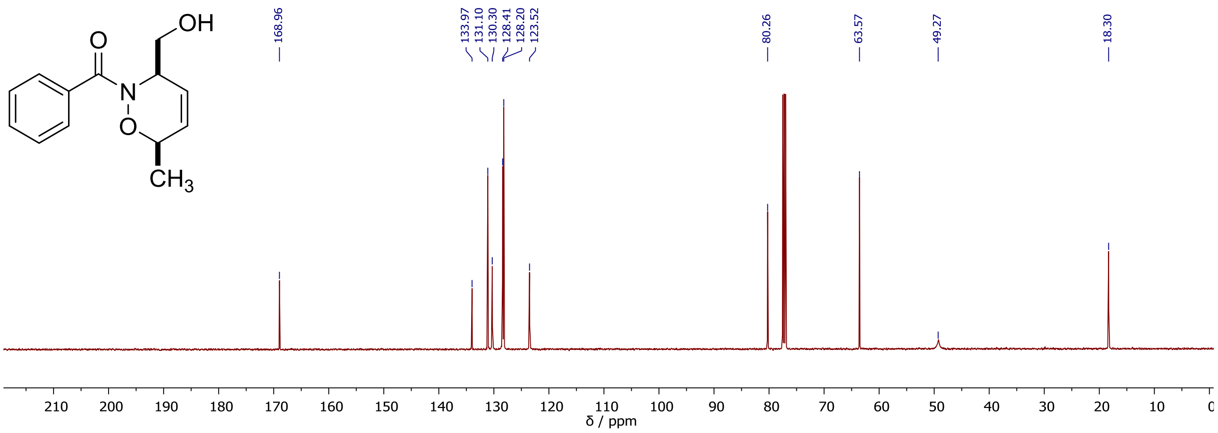

Figure S19: ^{13}C NMR spectrum of *cis*-(3-(hydroxymethyl)-6-methyl-3,6-dihydro-2*H*-1,2-oxazin-2-yl)(phenyl)methanone (**3k**) in $CDCl_3$ at 126 MHz and rt.

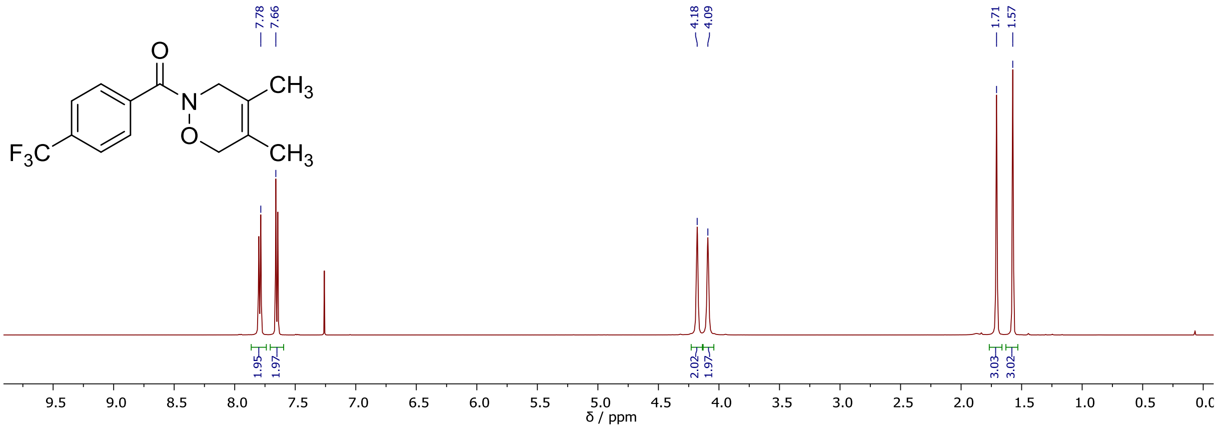

Figure S20: ^{1}H NMR spectrum of (4,5-Dimethyl-3,6-dihydro-2*H*-1,2-oxazin-2-yl)(4-(trifluoromethyl)phenyl)methanone (**5a**) in $CDCl_3$ at 500 MHz and rt.

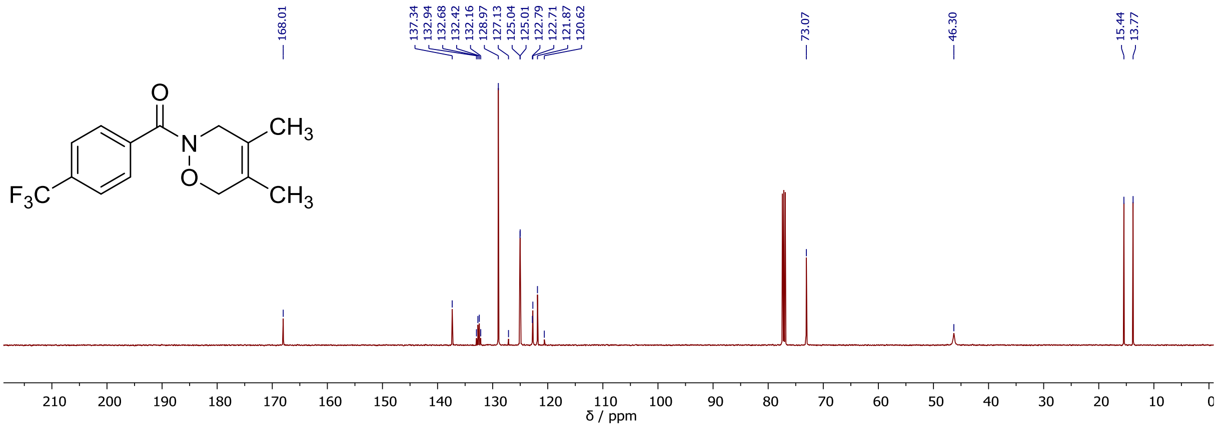

Figure S21: ^{13}C NMR spectrum of (4,5-Dimethyl-3,6-dihydro-2*H*-1,2-oxazin-2-yl)(4-(trifluoromethyl)phenyl)methanone (**5a**) in $CDCl_3$ at 126 MHz and rt.

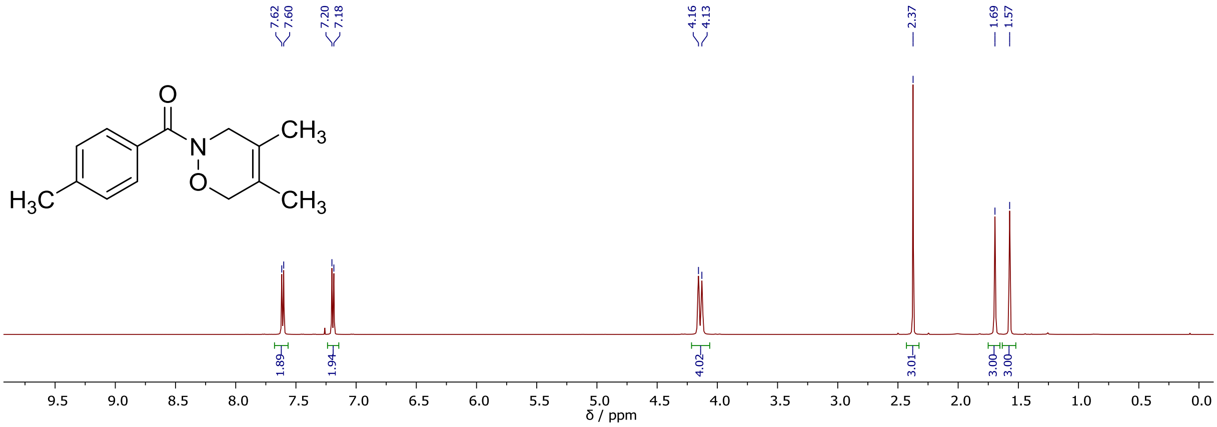

Figure S22: ^{1}H NMR spectrum of (4,5-dimethyl-3,6-dihydro-2*H*-1,2-oxazin-2-yl)(*p*-tolyl)methanone (**6a**) in $CDCl_3$ at 500 MHz and rt.

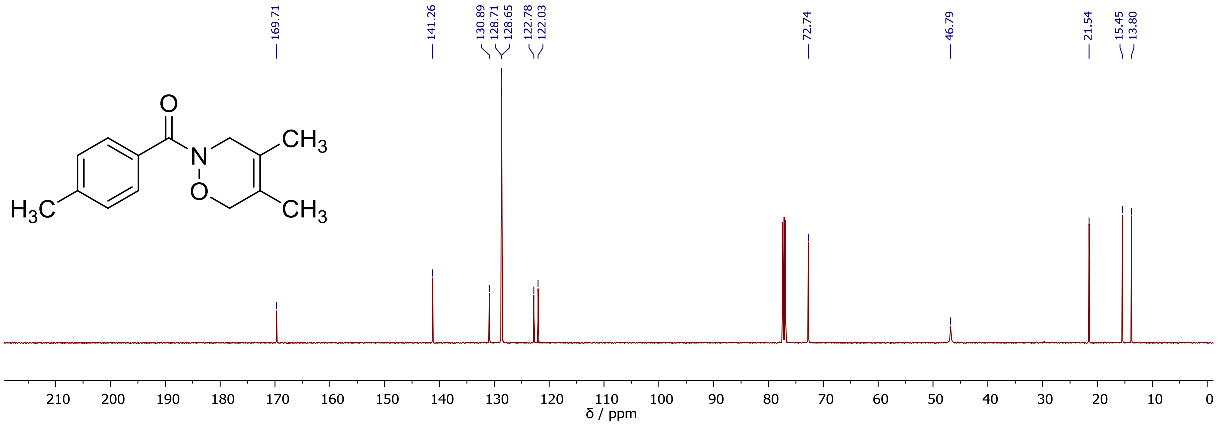

Figure S23: ^{13}C NMR spectrum of (4,5-dimethyl-3,6-dihydro-2*H*-1,2-oxazin-2-yl)(*p*-tolyl)methanone (**6a**) in $CDCl_3$ at 126 MHz and rt.

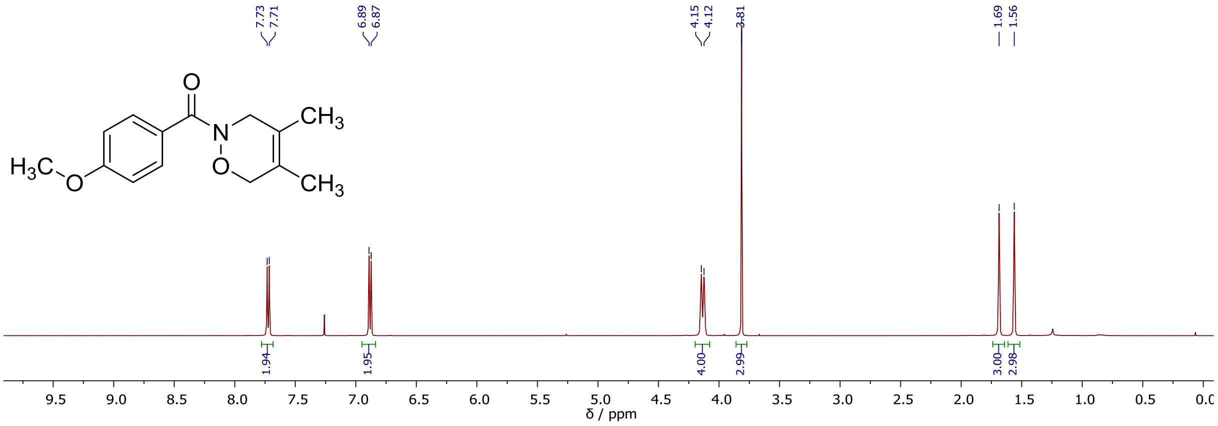

Figure S24: ^{1}H NMR spectrum of (4,5-dimethyl-3,6-dihydro-2*H*-1,2-oxazin-2-yl)(4-methoxyphenyl)methanone (**7a**) in $CDCl_3$ at 500 MHz and rt.

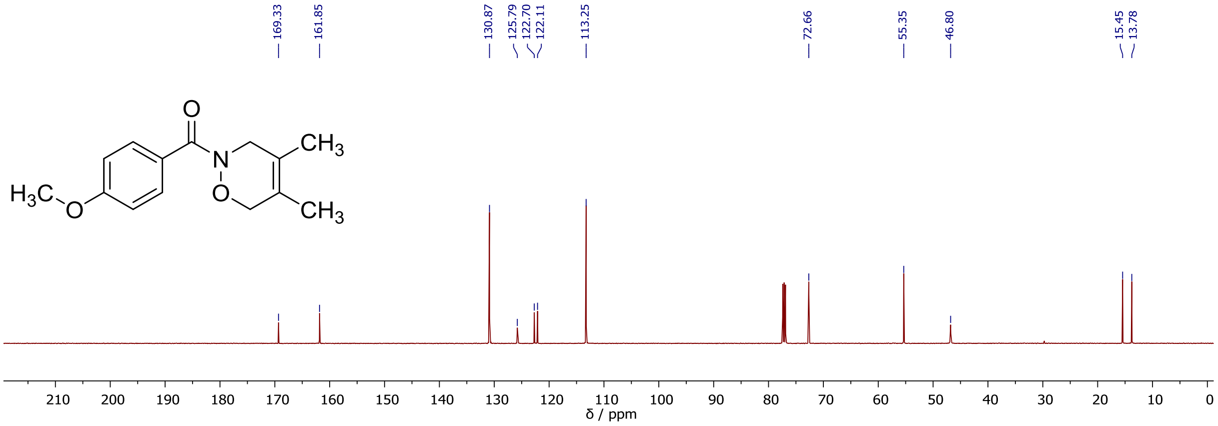

Figure S25: ^{13}C NMR spectrum of (4,5-dimethyl-3,6-dihydro-2*H*-1,2-oxazin-2-yl)(4-methoxyphenyl)methanone (**7a**) in $CDCl_3$ at 126 MHz and rt.

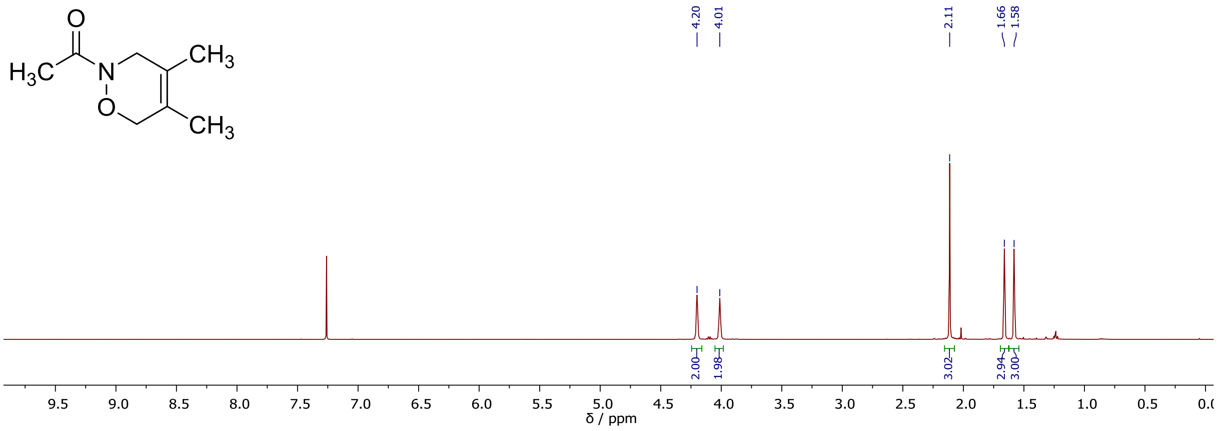

Figure S26: ^{1}H NMR spectrum of 1-(4,5-dimethyl-3,6-dihydro-2*H*-1,2-oxazin-2-yl)ethan-1-one (**8a**) in $CDCl_3$ at 500 MHz and rt.

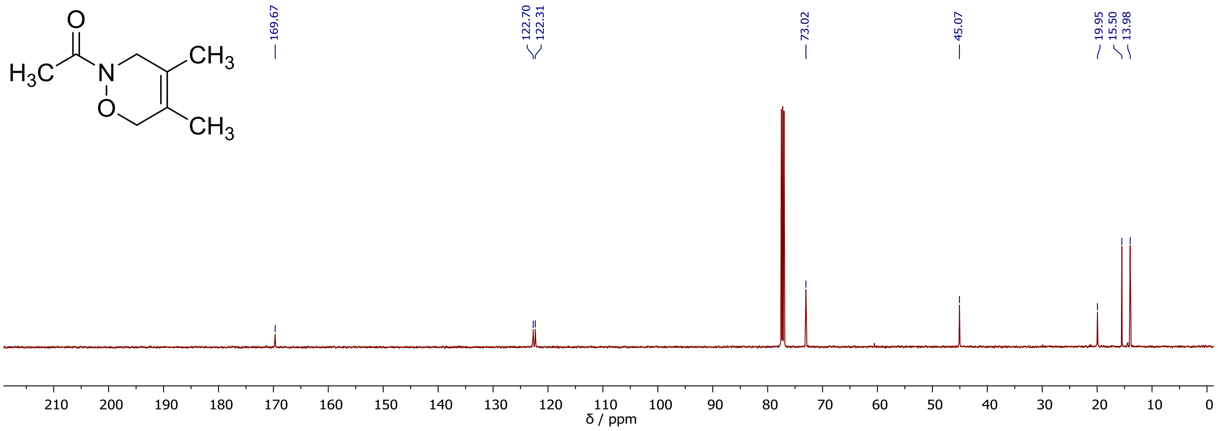

Figure S27: ^{13}C NMR spectrum of 1-(4,5-dimethyl-3,6-dihydro-2*H*-1,2-oxazin-2-yl)ethan-1-one (**8a**) in $CDCl_3$ at 126 MHz and rt.

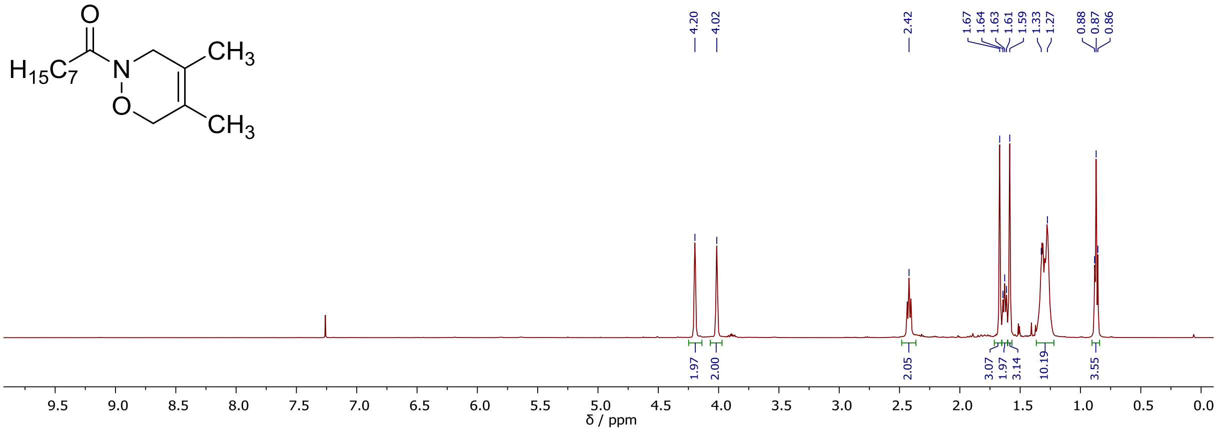

Figure S28: ^{1}H NMR spectrum of 1-(4,5-dimethyl-3,6-dihydro-2*H*-1,2-oxazin-2-yl)octan-1-one (**9a**) in $CDCl_3$ at 500 MHz and rt.

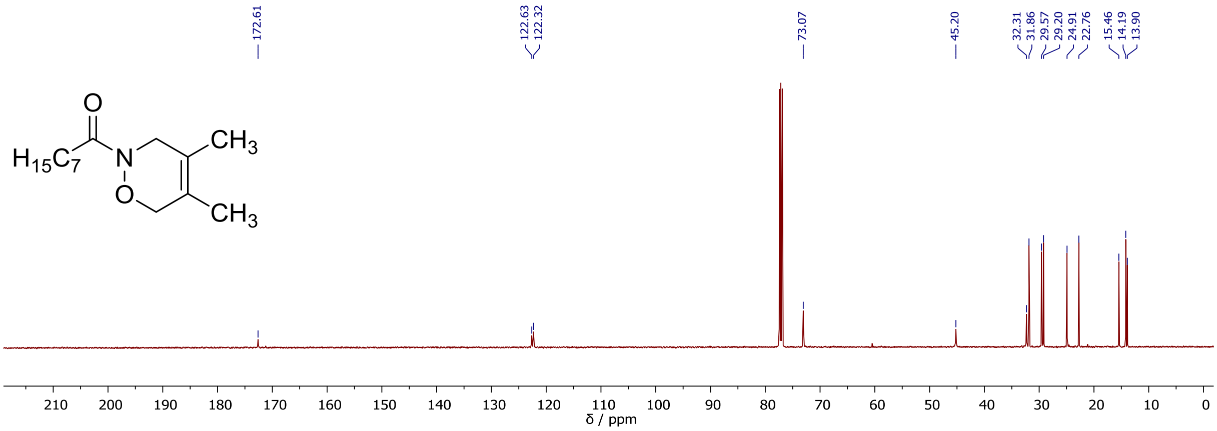

Figure S29: ^{13}C NMR spectrum of 1-(4,5-dimethyl-3,6-dihydro-2*H*-1,2-oxazin-2-yl)octan-1-one (**9a**) in $CDCl_3$ at 126 MHz and rt.

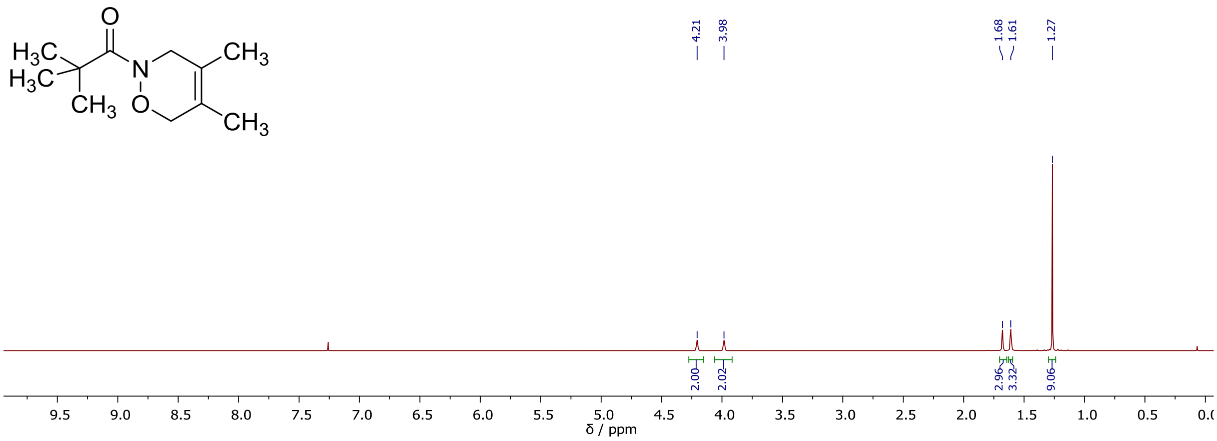

Figure S30: ^{1}H NMR spectrum of 1-(4,5-dimethyl-3,6-dihydro-2*H*-1,2-oxazin-2-yl)-2,2-dimethylpropan-1-one (**10a**) in $CDCl_3$ at 500 MHz and rt.

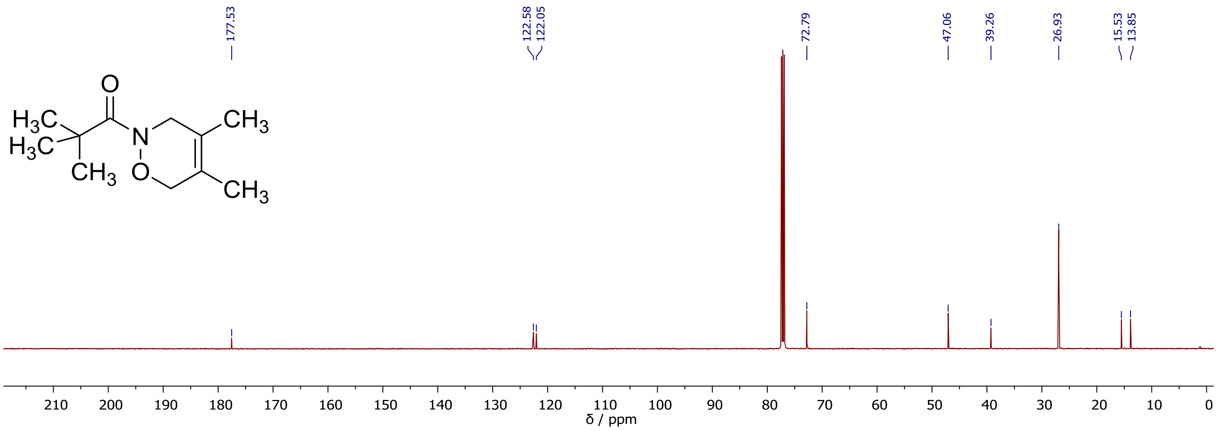

Figure S31: ^{13}C NMR spectrum of 1-(4,5-dimethyl-3,6-dihydro-2*H*-1,2-oxazin-2-yl)-2,2-dimethylpropan-1-one (**10a**) in $CDCl_3$ at 126 MHz and rt.

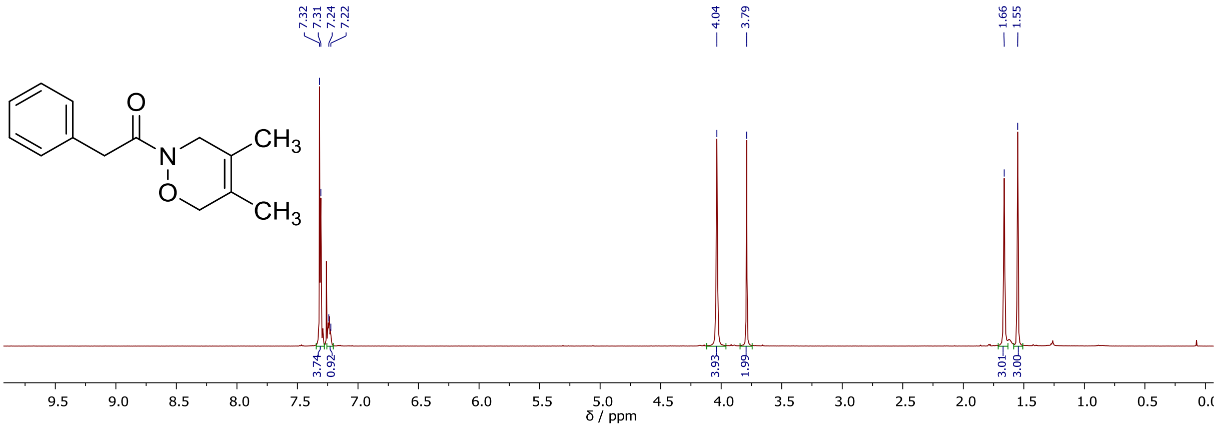

Figure S32: ^{1}H NMR spectrum of 1-(4,5-dimethyl-3,6-dihydro-2*H*-1,2-oxazin-2-yl)-2-phenylethan-1-one (**11a**) in $CDCl_3$ at 500 MHz and rt.

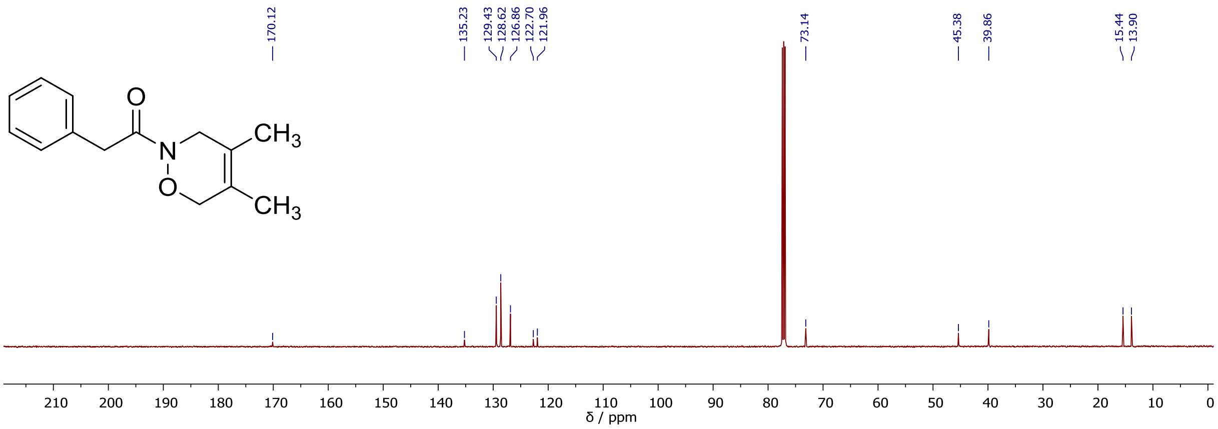

Figure S33: ^{13}C NMR spectrum of 1-(4,5-dimethyl-3,6-dihydro-2*H*-1,2-oxazin-2-yl)-2-phenylethan-1-one (**11a**) in $CDCl_3$ at 126 MHz and rt.

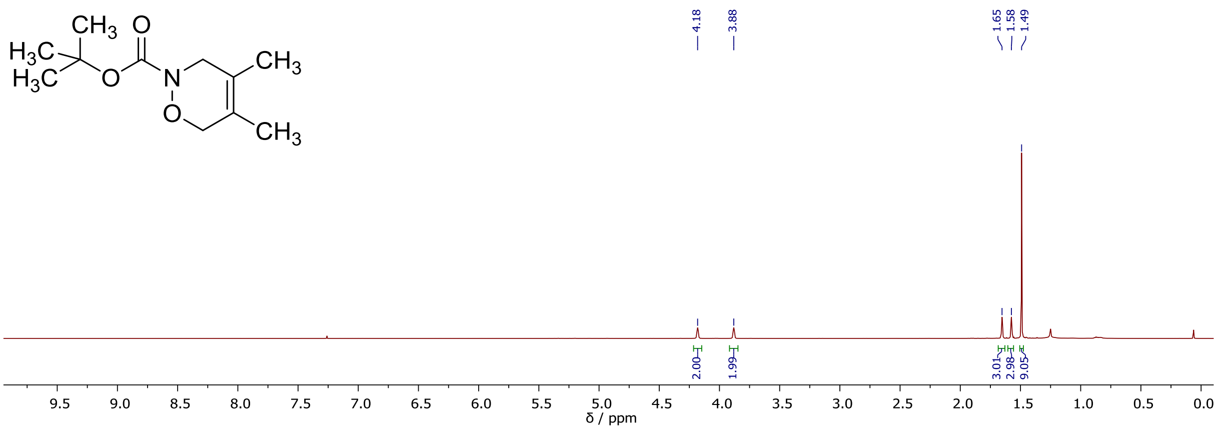

Figure S34: ^{1}H NMR spectrum of *tert*-butyl 4,5-dimethyl-3,6-dihydro-2*H*-1,2-oxazine-2-carboxylate (**12a**) in $CDCl_3$ at 500 MHz and rt.

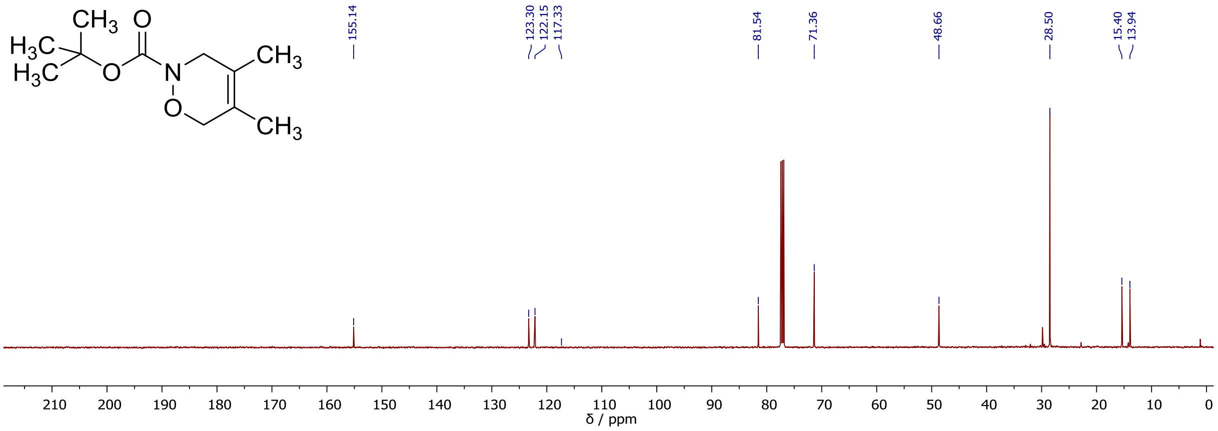

Figure S35: ^{13}C NMR spectrum of *tert*-butyl 4,5-dimethyl-3,6-dihydro-2*H*-1,2-oxazine-2-carboxylate (**12a**) in $CDCl_3$ at 126 MHz and rt.

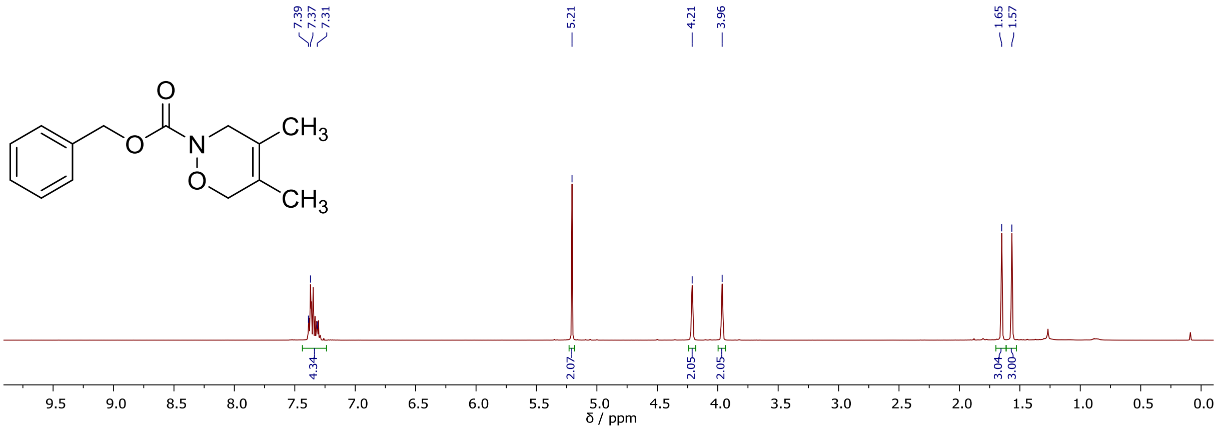

Figure S36: ^{1}H NMR spectrum of benzyl 4,5-dimethyl-3,6-dihydro-2*H*-1,2-oxazine-2-carboxylate (**13a**) in $CDCl_3$ at 500 MHz and rt.

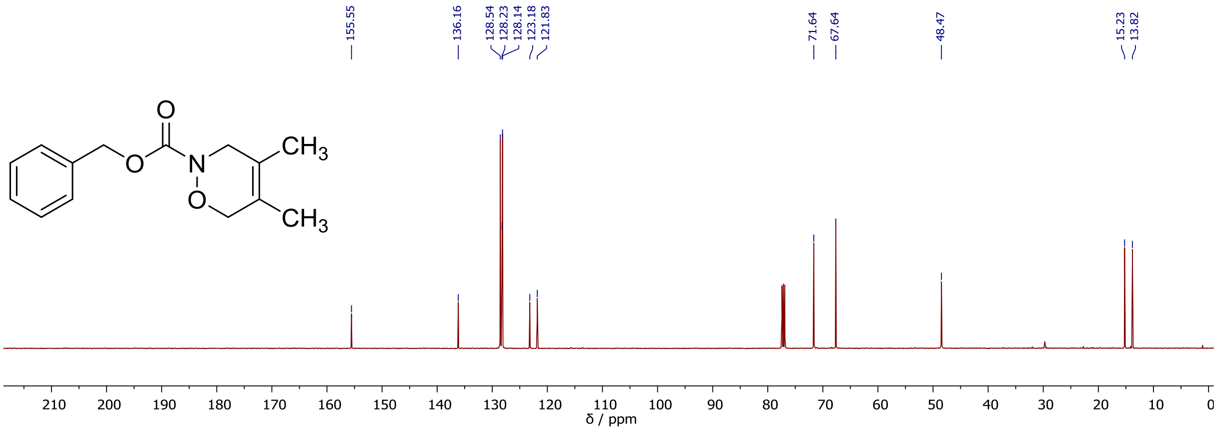

Figure S37: ^{13}C NMR spectrum of Benzyl 4,5-dimethyl-3,6-dihydro-2*H*-1,2-oxazine-2-carboxylate (**13a**) in $CDCl_3$ at 126 MHz and rt.

6.4 NMR spectrum of the crude reaction product

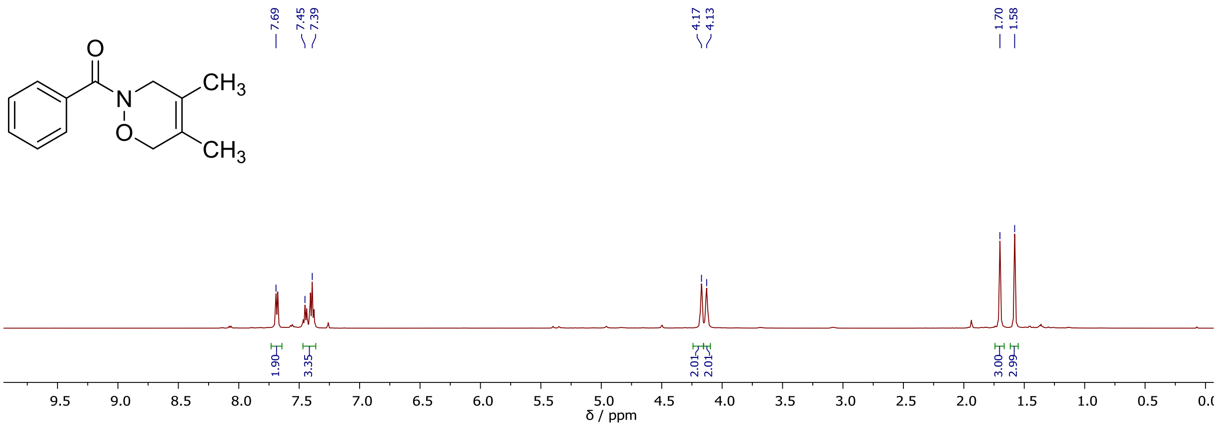

Figure S38: ^{1}H NMR spectrum of the crude reaction product (4,5-dimethyl-3,6-dihydro-2*H*-1,2-oxazin-2-yl)(phenyl)methanone (**3a**) in $CDCl_3$ at 500 MHz and rt.

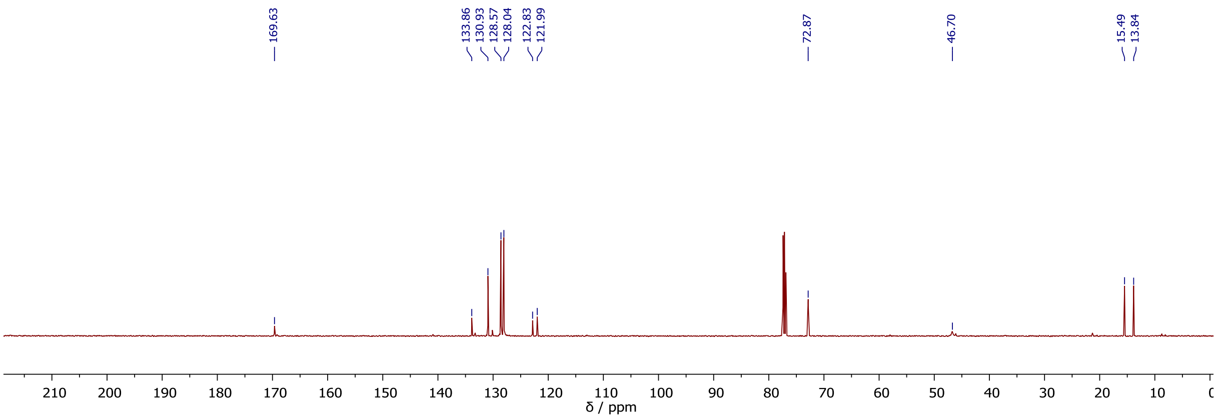

Figure S39: ^{13}C NMR spectrum of the crude reaction product (4,5-dimethyl-3,6-dihydro-2*H*-1,2-oxazin-2-yl)(phenyl)methanone (**3a**) in $CDCl_3$ at 500 MHz and rt.

7 Mechanistic investigations

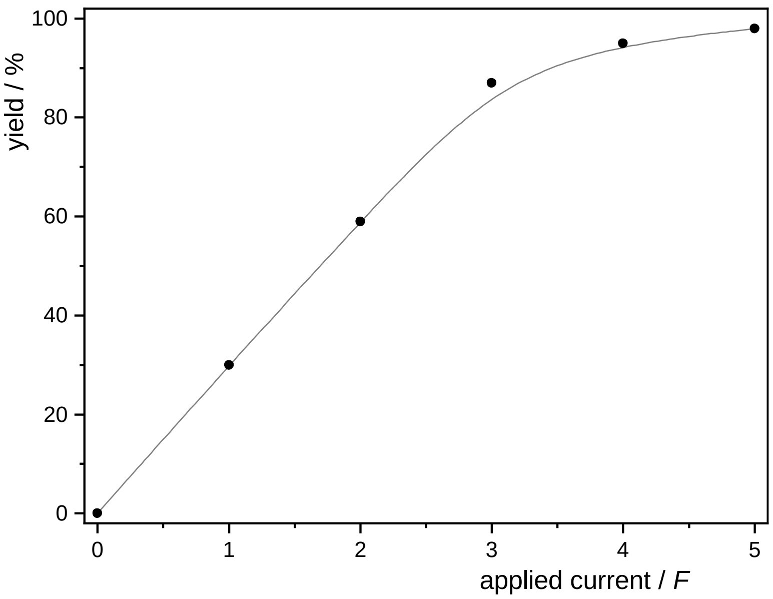

Figure S40: Determined correlation between the applied current and the product yield of the optimized reaction.

7.1 Cyclic voltammetry

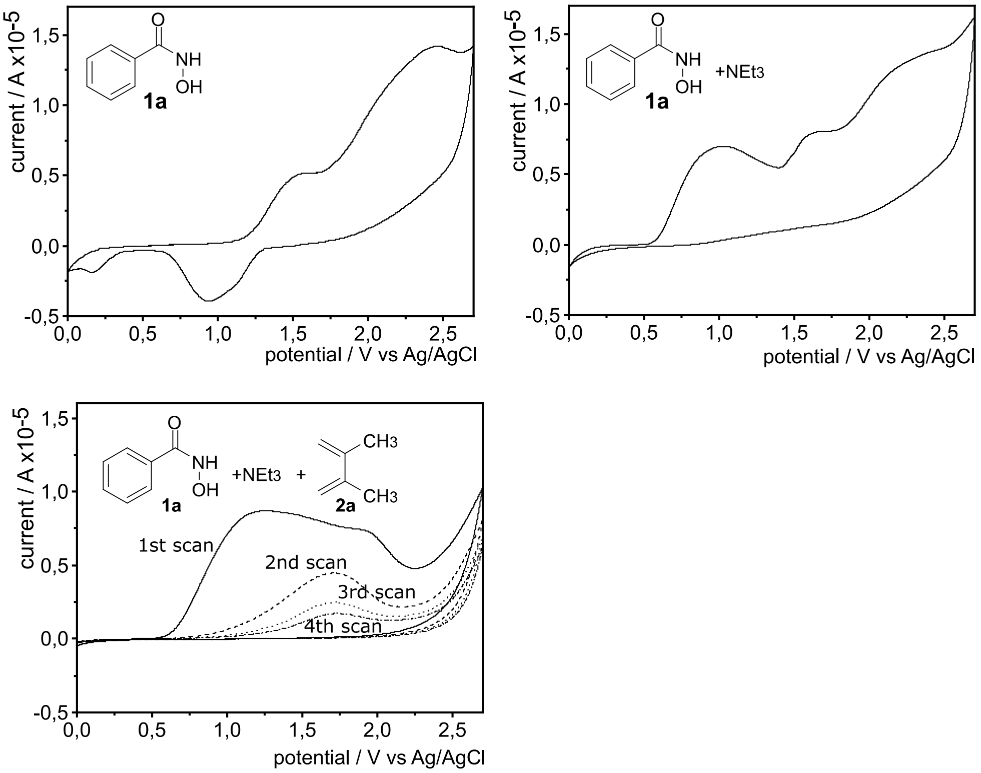

Figure S41: Cyclic voltammograms of **1a**/**2a**/NEt_3 in a 0.2 M solution of *n*Bu_4NBF_4 in 8 mL DCM + 2 mL HFIP at a scan rate of 0.1 V/s at a platinum working electrode (2.0 mm diameter).

8 References

[1] L. Pitzer, F. Schäfers, F. Glorius, *Angew. Chem*. **2019**, *131*, 8660–8664; *Angew. Chem. Int. Ed*. **2019**, *58*, 8572–8576.

[2] K. N. Zelenin, I. A. Motorina, L. A. Sviridova, I. P. Bezhan, A. Y. Ershov, G. A. Golubeva, Y. G. Bundel, *Chem. Heterocycl. Compd*. **1987**, *23*, 1018–1024.

[3] E. S. Aldeen, T. Elsaman, M. S. Mohamed, M. E. Adam, M. A. Mohamed, *Asian J. Chem.* **2019**, *31*, 181–185.

[4] G. Dettori, S. Gaspa, A. Porcheddu, L. D. Luca, *Adv. Synth. Catal*. **2014**, *356*, 2709–2713.
